# Insights into the Imprinting and Rebinding Performance of Molecularly Imprinted Hybrids for Bisphenol A and Bisphenol F

**DOI:** 10.1021/acsami.5c03038

**Published:** 2025-04-30

**Authors:** Kae-Zheng Chin, Sue-min Chang

**Affiliations:** † Institute of Environmental Engineering, National Yang Ming Chiao Tung University, 1001 University Road, Hsinchu 300093, Taiwan; ‡ Graduate Institute of Environmental Engineering, National Taiwan University, No. 1, Section 4, Roosevelt Road, Da’an District, Taipei 10617, Taiwan

**Keywords:** molecular imprinting, bisphenol adsorption, NOESY NMR analyses, noncovalent forces, binding interaction

## Abstract

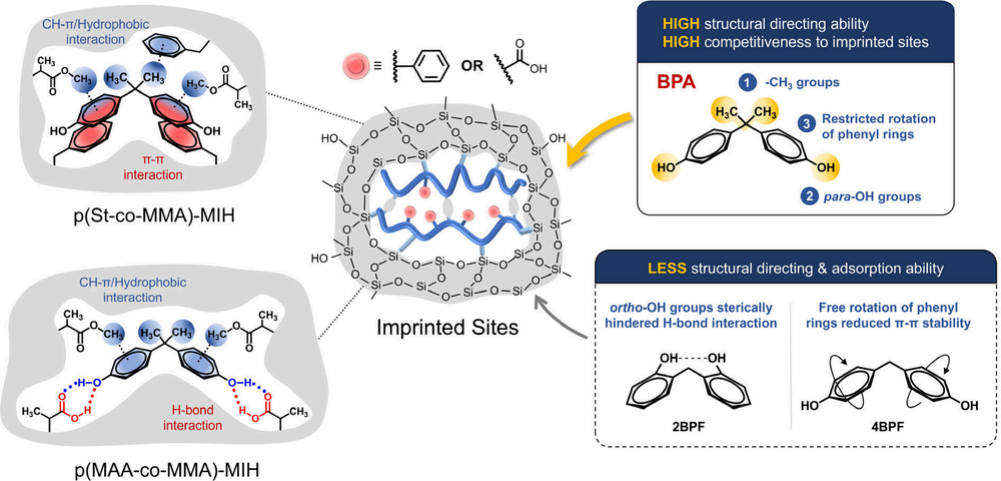

This study investigates the factors influencing the imprinting performance of molecularly imprinted hybrids (MIHs) with various template/monomer associations and their corresponding adsorption ability for three bisphenol analogues, bisphenol A (BPA), 2,2′-bisphenol F (2BPF), and 4,4′-bisphenol F (4BPF). Styrene (St) and methacrylic acid (MAA) were selected as the primary functional monomers for template complexation. Compared with hydrophilic MAA monomers, hydrophobic St monomers were more favorable for BPA imprinting, despite the lower binding energy of π–π interactions compared to hydrogen bonds. However, St monomers were unsuitable for 4BPF imprinting, while 2BPF exhibited limited complexation with MAA monomers. Among the bisphenols, BPA demonstrated the strongest imprinting capability, leading MIHs to exhibit the highest imprinting factor (IF = 14–18), adsorption capacity (*Q*_max_ = 43.7–47.6 mg/g), binding affinity (*K*_L_ = 4.52–6.74 L/mg, Δ*H*_ads_^°^ = −35.2 to −38.9 kJ/mol, and Δ*S*_ads_^°^ = −40.5 to −50.6 J mol^–1^ K^–1^), and selectivity over 2BPF and 4BPF (2.0–3.5). In contrast, 2BPF- and 4BPF-imprinted hybrids exhibited significantly lower adsorption capacities (*Q*_max_ = 19.4–26.7 mg/g) and binding affinities (*K*_L_ = 1.22–4.35 L/mg) for their respective templates. In competitive adsorption systems, bisphenol rebinding followed the trend BPA > 2BPF > 4BPF, regardless of which template was used for imprinting. Based on NMR analysis, the superior structure-directing and competitive rebinding abilities of BPA are attributed to the restricted rotation of its two phenyl groups, *p*-OH groups, and additional -CH_3_ groups on the bridged carbon, which enhance π–π stacking, H-bond, CH−π, and hydrophobic interactions within the imprinted cavities. In contrast, the *o*-OH groups of 2BPF and the rotational phenyl groups of 4BPF hinder their imprinting and rebinding via H-bond and π–π interactions, respectively.

## 1 Introduction

Bisphenol A (BPA) and its substitute, bisphenol F (BPF), widely found in various consumer products, are known for their adverse effects as endocrine-disrupting chemicals.^1−3^ To address their impact on human health and the environment, there is an increasing demand for advanced adsorbents to effectively separate and concentrate these compounds in water. Molecular imprinting is a promising approach to create artificial recognition materials exhibiting high binding affinity and selectivity for analytes of interest.^4−7^ This method involves binding template molecules with functional monomers, cross-linking the template/monomer complexes to form a three-dimensional polymeric matrix, and subsequently removing the incorporated template from the resulting polymers. Understanding of the molecular interactions within self-assembled imprinting systems is crucial for designing adsorbents with the desired adsorption performance. While molecular imprinting has been extensively employed to develop adsorbents for various applications, fundamental research on the factors governing imprinting effectiveness and resulting adsorption ability and selectivity among structural analogues remains limited, leaving some issues unresolved.

Effective imprinting relies on stable template/monomer complexes.^8^ Due to simplicity and reversibility, noncovalent bonds are generally employed to complex templates with functional monomers. Monomers containing -COOH, -CONH_2_, pyridine, or aromatic functional groups, such as methacrylic acid (MAA),^9,10^ acrylamide (AAM),^11^ 4-vinylpyridine (4-VP),^12^ and styrene (St),^13^ are commonly selected to establish H-bonds or π–π interactions with bisphenols. The binding energies of these functional monomers to BPA are calculated to be 10.16–13.61 kcal/mol for H-bonds, while the π–π stacking energy associated with St is 0.7–3.0 kcal/mol.^14−16^ Compared to π–π interactions, stronger H-bonds are considered more advantageous for imprinting efficiency. However, this benefit may be offset by the formation of H-bonded dimers between monomers, which can hinder their interaction with templates.^17^ Additionally, template rebinding via H-bonds may be interfered in aqueous environments.^5^ In contrast, the hydrophobicity of St moieties in polymers facilitates the partitioning of bisphenols into the imprinted matrix.^18^ Furthermore, despite the lower binding energies of π–π interactions, St monomers have demonstrated high imprinting performance and strong adsorption selectivity for BPA.^19^ To date, the contributions of H-bonds and π–π interactions to the imprinting of bisphenols have not yet been systematically compared and evaluated. Moreover, binding energies and imprinting performance vary among bisphenol analogues for a given functional monomer.^20,21^ Since both H-bonds and π–π interactions are orientation-dependent, the variation is associated with template molecular configurations. However, the limitation of these two binding mechanisms in imprinting, as well as the structural directing ability of bisphenol analogues concerning the analogue molecular configurations, has not yet been clarified.

Although imprinted cavities exhibit strong exclusion ability for compounds with sizes and shapes differing from the templates, the customized binding sites can also accommodate structural analogues to a certain extent.^22^ Based on this characteristic, dummy-template molecularly imprinted polymers (DMIPs) have been developed using compounds with similar structural features and chemical attributes to the target analytes as alternative templates to avoid positive bias caused by the release of template residues when the DMIPs are used to enrich analytes for trace-level analysis.^23^ DMIPs have demonstrated their ability to recognize target molecules as effectively as conventional imprinted polymers. In some cases, they even exhibit greater specificity for the target compared to the dummy template, in particular, as observed in the adsorption of BPA by DMIPs prepared using tetrabromobisphenol A (TBBPA) or 4,4′-bisphenol F (4BPF) as dummy templates.^24−28^ The mechanisms underlying this distinct selectivity for the target analytes over the dummy templates remain unclear.

**Figure 1:**
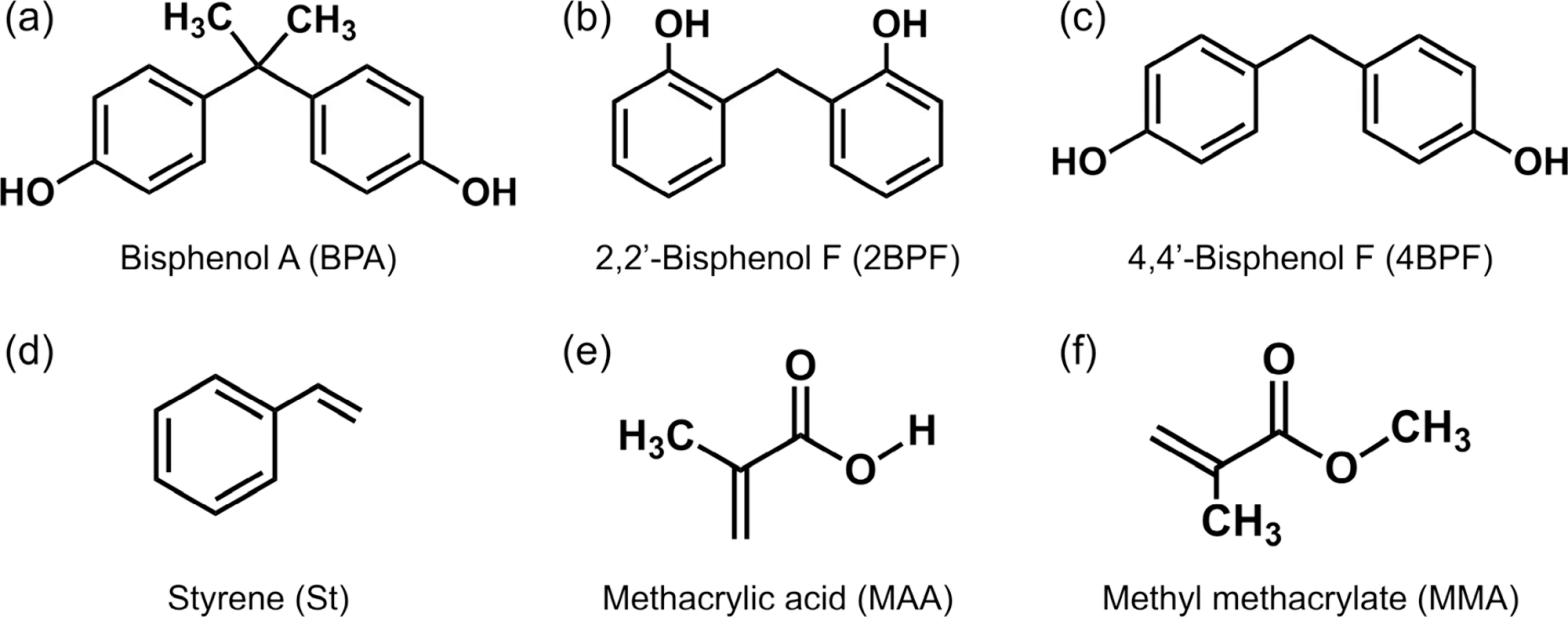
Chemical structures of templates (a) BPA, (b) 2BPF, and (c) 4BPF and monomers (d) St, (e) MAA, and (f) MMA used in this study.

In this study, we aimed to investigate the factors influencing the imprinting and rebinding of three bisphenols, BPA, 2,2′-bisphenol F (2BPF), and 4BPF, in an organic–inorganic hybrid system with a focus on template molecular configurations, hydrophilicity/hydrophobicity of functional monomers, and compatible intermolecular interactions between the template and monomers/polymers through π–π interactions or H-bonds. Previously, we developed a molecularly imprinted hybrid (MIH) targeting BPA by cross-linking styrene (St)/methyl methacrylate (MMA) copolymers with SiO_2_ clusters.^7^ The MIH exhibited an impressive BPA adsorption capacity (40.3 mg/g), a substantial imprinting factor (14), and remarkable selectivity. In this study, the MIH was used as a model system to further explore imprinting characteristics and guest–host interactions with different structural analogues and functional monomers. The three bisphenol analogues, BPA, 2BPF, and 4BPF, as illustrated in Figure 1, were used as templates. Two primary functional monomers, St and MAA, were employed to interact with the templates through π–π interactions and H-bonds, respectively, while MMA monomers were used to form linear copolymer chains that encapsulated the template and shaped imprinted cavities along the template conformation. The adsorption ability, thermodynamic parameters, and selectivity of the MIHs/non-imprinted hybrids (NIHs) were systematically analyzed. Moreover, one- and two-dimensional ^1^H nuclear magnetic resonance (NMR) spectroscopy was utilized to determine the interactions between the monomers/copolymers and the templates at molecular levels. By cross-comparing the adsorption and NMR results, optimal template/monomer combinations for achieving high adsorption capacities were identified. The key molecular features of the templates and functional monomers that determined their superiority or limitations in imprinting and adsorption were clarified. Moreover, guidelines for selecting suitable functional monomers based on the molecular configuration of templates to achieve the desired adsorption performance were established.

## 2 Experimental Section

### 2.1 Preparation of Molecularly Imprinted Hybrids

**Scheme 1:**
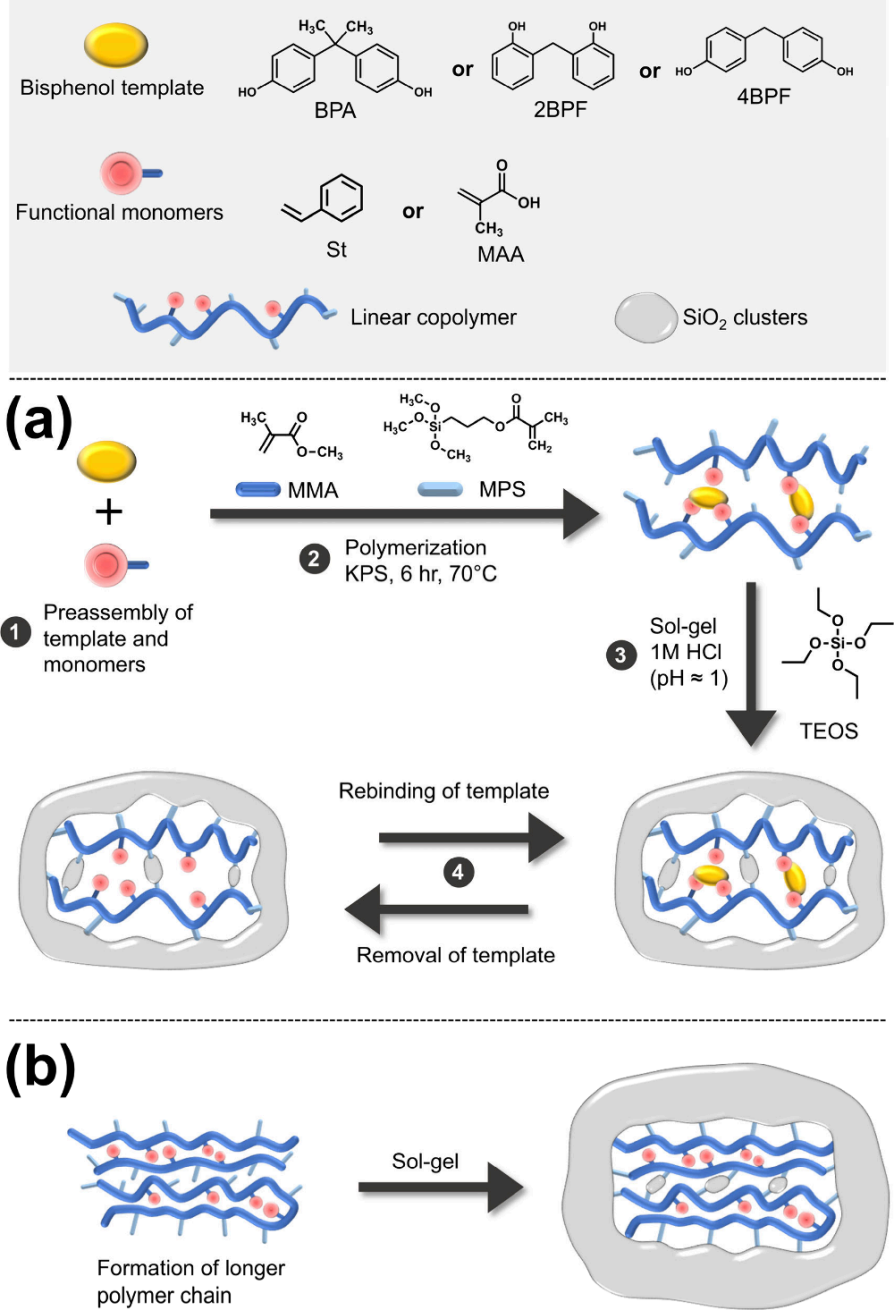
(a) Schematic Diagram of the Preparation Procedures for MIHs and the Resulting Imprinting Structure and (b) Cross-Linking of Long Polymer Chains with SiO_2_ Clusters during the Synthesis of NIHs In the absence of template hindrance, longer polymer chains are formed, resulting in larger particle sizes compared to MIHs. Additionally, stronger interactions among the extended polymer chains drive more SiO_2_ clusters to deposit on the particle exterior, leading to a denser structure in NIHs, in contrast to the looser, nanoporous structure in MIHs.

The synthesis of MIHs followed the procedures outlined in our previous study^7^ and is illustrated in Scheme 1. BPA (99%, Tokyo Chemical Industry Co., Ltd.), 2BPF (98%, Sigma-Aldrich), and 4BPF (98%, Alfa Aesar) were employed as templates. St (>99%, Sigma-Aldrich) and MAA (99%, Thermo) were used as primary functional monomers to form complexes with templates, while MMA (99%, Alfa Aesar) was used to form linear polymeric chains. A silane coupling agent, 3-(methacryloxyl) propyltrimethoxysilane (MPS, 97%, Alfa Aesar), was used to covalently bond polymer chains and SiO_2_, and tetraethyl orthosilicate (TEOS, 98%, Seedchem) was used as the precursor of the inorganic cross-linker, SiO_2_. The compositions of the templates and monomers for different samples are listed in Table 1. The template and functional monomer at desired amounts were dissolved in 6.0 mL of anhydrous ethanol (99.5%, ECHO) under agitation for 20 min, followed by the addition of MMA and MPS (5 mmol). Polymerization was initiated by adding 1.0 mL of a potassium persulfate (KPS, 99%, Sigma-Aldrich) aqueous solution at 70 °C, with the concentration of the KPS solution adjusted to ensure that the added KPS amount was 1.0 mol % of the total monomers. Polymerization proceeded at 70 °C for 6 h. Subsequently, TEOS (15 mmol), 0.9 mL of DI water, and 1.0 mL of a 1.0 M HCl solution were added to the solution to cross-link the polymer chains by forming SiO_2_ clusters via sol–gel reactions. After 2 h reactions, the colloidal solution was transferred into a crucible and dried at 50 °C overnight, and the obtained solid products were ground into fine powders. The templates were then extracted from the MIH powders with methanol using a microwave extraction method. The extraction was conducted at 75 °C for 20 min and repeated 10 times to ensure complete removal of the template. For comparison, non-imprinted hybrids (NIHs) were also prepared using identical recipes and procedures as described above but without the addition of templates. The morphologies, textures, and particle sizes of MIHs and NIHs were characterized by scanning electron microscopy (SEM), transmission electron microscopy (TEM), N_2_ adsorption–desorption, and dynamic light scattering (DLS), as detailed in the Supporting Information. Additionally, the minimal release of BPA from a St-based MIH after extraction to a methanol/acetic acid (9:1 (v/v)) eluent was analyzed using high-performance liquid chromatography-tandem mass spectrometry (HPLC-MS/MS) to ensure a negligible amount of the template remained in the MIH.

**Table 1:** Chemical Compositions for the Different Types of MIHs

	template (mmol)	primary functional monomer (mmol)		secondary functional monomer (mmol)	silane coupling agent (mmol)	inorganic cross-linker (mmol)
MIH	BPA/2BPF/4BPF	St	MAA	MMA	MPS	TEOS
St-based	1	2	–a	5	5	15
MAA-based	1	–a	2	5	5	15
MMA-based	1	–a	–a	7	5	15

a Not involved.

### 2.2 Adsorptions of Bisphenols

The binding affinity of MIHs for their templates was evaluated by conducting adsorption isotherms at 298, 308, 318, and 328 K. Each MIH (15 mg) was separately dispersed in 15 mL of a single-analyte aqueous solution (BPA, 2BPF, or 4BPF) at varying initial concentrations (10–100 mg/L). The suspensions were magnetically stirred for 2 h of adsorption, after which the adsorbents were removed by centrifugation. The concentration of unbound analytes in supernatants was determined using a UV–vis spectrometer (Hitachi 3010) based on the absorbance at 276, 272, and 275 nm for BPA, 2BPF, and 4BPF, respectively. The equilibrium adsorption amounts (*Q*_e_, mg/g) for the analytes were calculated using eq 1.
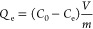
1where *C*_0_ (mg/L) is the initial concentration of analytes, *C*_e_ (mg/L) is the equilibrium concentration of unbound analytes, *V* (mL) is the volume of the solution, and *m* (mg) is the weight of MIHs or NIHs. The imprinting factor (IF) is defined as the ratio between the *Q*_e_ of a MIH and its corresponding NIH (eq 2).
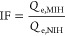
2

The isotherms obtained at different temperatures were fitted with the Langmuir model to determine the Langmuir constant (*K*_L_). Thermodynamic parameters, including Δ*G*_ads_^°^, Δ*H*_ads_^°^, and Δ*S*_ads_^°^, were further derived based on the *K*_L_ values.

### 2.3 Selective Adsorptions

The molecular recognition ability of MIHs and NIHs was assessed through competitive adsorption in aqueous solutions containing BPA, 2BPF, and 4BPF, with each bisphenol at an initial concentration of 20 mg/L. Equal amounts (20 mg) of MIHs or NIHs were separately suspended in 20 mL of the mixture under agitation. Following a 2 h adsorption period, the remaining concentrations of the bisphenols in the supernatants were analyzed using an HPLC instrument equipped with a photodiode array detector (HPLC-PDA, Waters 2695/2996). For HPLC analysis, 20 μL of supernatants was injected into the chromatography system, and the bisphenol analytes were separated using a hypersil BDS C18 column (Thermo, 4.6 mm × 250 mm, 5 μm particle size) with a mobile phase consisting of water/acetonitrile (50:50, v/v) at a flow rate of 1 mL/min. The absorbances of BPA and 4BPF were measured at 277 nm, while that of 2BPF was measured at 273 nm. The retention times of 4BPF, BPA, and 2BPF were 4.4, 5.7, and 6.7 min, respectively. The selective binding of MIHs for the analogues is determined by the relative selectivity coefficient (*k*′), as shown in eq 3, where *k*′ is derived from the selectivity coefficient (*k*, eq 4) and the distribution coefficient (*K*_d_, eq 5).^29^
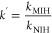
3
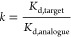
4
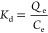
5where *Q*_e_ (mg/g) represents the adsorbed amount of the target analyte or analogue and *C*_e_ (mg/L) denotes the remaining concentration in the supernatant at equilibrium.

### 2.4 Studies of Complex Formation by ^1^H NMR Spectroscopy

The interactions between the templates and functional monomers were examined using a 600 MHz NMR spectrometer (VARIAN VNMRS-600). One-dimensional ^1^H NMR analysis and two-dimensional ^1^H–^1^H nuclear Overhauser effect spectroscopy (NOESY) were conducted to explore the intermolecular interactions between bisphenols and functional monomers. For the experiments, 1.0 mmol of each template (BPA, 2PBF, or 4BPF) was separately mixed with 2.0 mmol of a functional monomer (St, MAA, or MMA) in dry DMSO-*d*_6_ according to the stoichiometry used for the preparation of MIHs. Additionally, to investigate the intermolecular interaction between the bisphenols and copolymers, including p(St-*co*-MMA), p(MAA-*co*-MMA), and pMMA, 2.0 mg of each template was individually mixed with 15 mg of the polymers in CDCl_3_ or DMSO-*d*_6_. These samples were analyzed by two-dimensional NOESY and ^1^H selective rotating-frame Overhauser effect spectroscopy (one-dimensional ROESY). p(St-*co*-MMA) and p(MAA-*co*-MMA) were synthesized with 2.0 mmol of St and 2.0 mmol of MMA and with 10 mmol of MMA and 10.0 mmol of MMA, respectively, while pMMA was synthesized with 10 mmol of MMA. Each synthesis was conducted by dissolving the monomers in 6.0 mL of anhydrous ethanol under stirring. Similar to the preparation for the MIHs, polymerization was initiated by adding 1.0 mL of a KPS aqueous solution (1.0 mol % of the total monomers) at 70 °C and continued for 6 h. The resulting polymers were precipitated by using methanol and separated from the suspensions by centrifugation. Subsequently, the obtained polymers were washed three times with methanol and then dried at 50 °C overnight.

## 3 Results and Discussion

### 3.1 Characteristics of MIHs and NIHs

**Figure 2:**
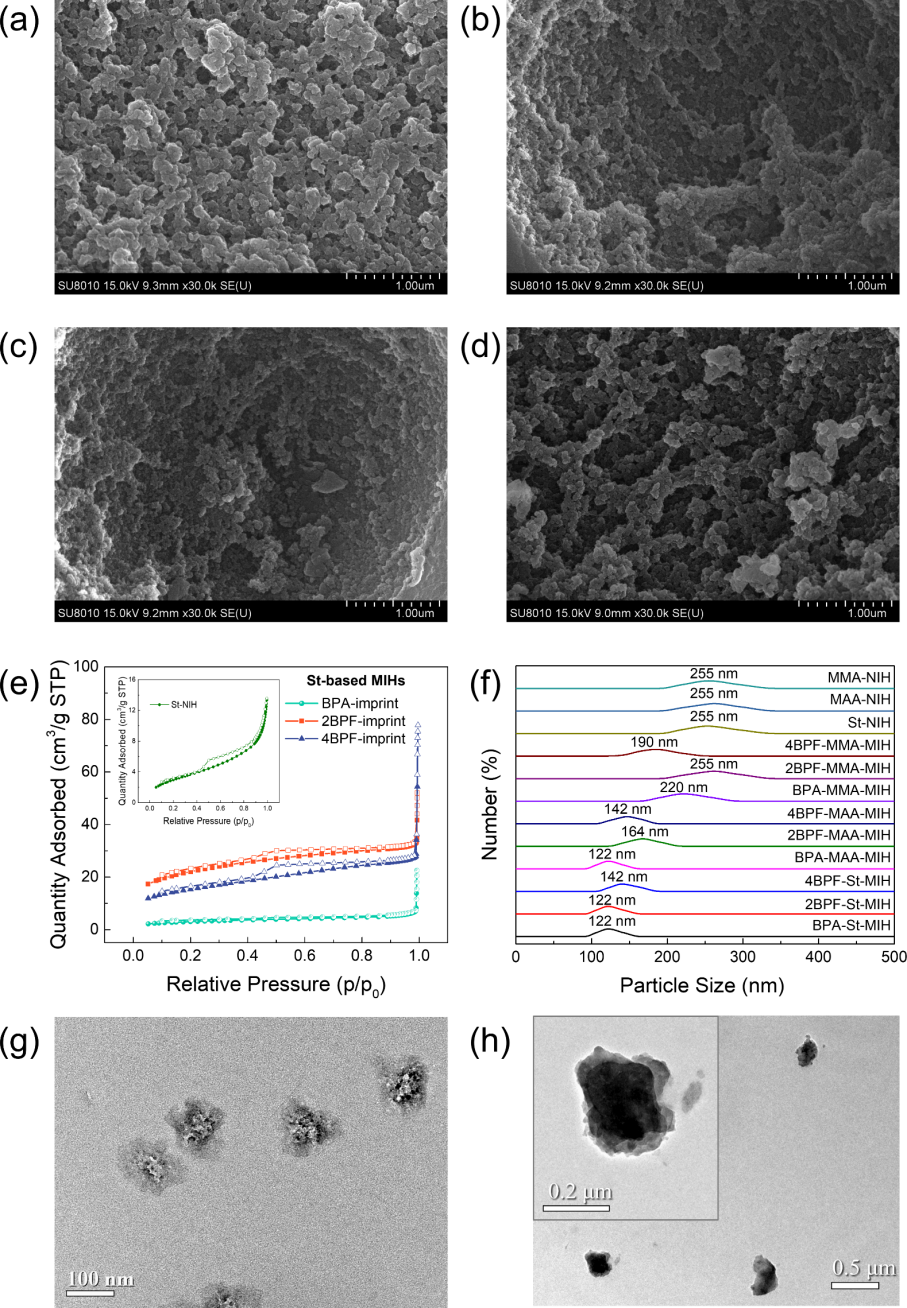
SEM images of St-based MIHs with the (a) BPA, (b) 2BPF, and (c) 4BPF imprints and (d) St-based NIH. (e) N_2_ adsorption–desorption isotherm curves of St-based MIHs and St-based NIH (inset). The filled symbol represents N_2_ adsorption, and the empty symbol represents N_2_ desorption. (f) Particle size distribution of each type of MIH and NIH in methanol. TEM images of (g) St-based MIH with BPA imprints and (h) St-based NIH. The inset in Figure 2h shows a higher magnification of a St-based NIH particle.

MIHs and NIHs were prepared through radical-induced polymerization and sol–gel reactions, as described in our previous work.^7^ Initially, linear polymer chains were formed through the polymerization between functional monomers and a coupling agent, while the monomers had formed complexes with templates. Subsequently, sol–gel reactions between the coupling agent and a Si-based precursor cross-linked the polymer chains, resulting in 3D imprinted nanostructures (Scheme 1). In our earlier work, the material properties, including the morphology, texture, particle size, composition, and surface charge, of a St-based MIH imprinted with BPA molecules and its corresponding NIH were thoroughly characterized.^7^ The roles of the organic framework and inorganic cross-linker in imprinting and adsorption ability were also identified. The MIH and NIH were found to contain 37–40 wt % organic components and 60–63 wt % SiO_2_ moieties. The organic polymer was primarily responsible for analyte binding, while the rigid SiO_2_ moiety confined the conformation of the imprinted cavities, minimized nonspecific adsorption, and improved water compatibility, which synergistically contributed to a high IF (14) and a high selectivity of the MIH. Successful removal of the imprinted molecules from the MIH via methanol extraction was confirmed by the disappearance of the characteristic template peak at 1515 cm^–1^ in FTIR spectra. In this study, this fact was further verified by a reduction in particle aggregation and a decrease in particle size after the extraction, as observed through SEM and DLS characterizations (Figure 2a and Figure S1a,b). Additionally, LC-MS/MS analysis of methanol eluents confirmed minimal BPA release from the extracted MIHs (0.1 ng/mL) (Supporting Information).

The morphologies, textures, and particle sizes of St-based, MAA-based, and MMA-based MIHs and NIHs were characterized by SEM, TEM, N_2_ adsorption–desorption, and DLS. Aggregates were observed in all MIHs and NIHs. For St-based MIHs, unclear grain boundaries were found in the BPA-imprinted hybrid due to stronger aggregation, whereas more distinct boundaries were noted in 2BPF- and 4BPF-imprinted hybrids (Figure 2a–c). Similarly, significant aggregation was observed in MAA- and MMA-based MIHs with BPA imprints (Figure S1c,d). In contrast to the MIHs, larger grain sizes were observed in the corresponding NIH powders (Figure 2d). Moreover, like BPA-imprinted hybrids, significant aggregation was noted in the NIH powders. The morphological features corresponded with the textural results. All of the MIHs and NIHs exhibited type II adsorption isotherms with H3 hysteresis loops (Figure 2e and Figure S2a–c), indicating slit-like pores. The pores, with a diameter of 3.6–4.1 nm, are attributed to voids between particles in the aggregates (Figure S2d–g). Due to significant aggregation, MIHs with BPA imprints showed small specific surface areas (SSAs) of 10.1–16.6 m^2^/g and small pore volumes of 0.011–0.033 cm^3^/g. In contrast, the 2BPF- and 4BPF-imprinted hybrids exhibited larger SSAs (27.8–99.0 m^2^/g) with larger pore volumes (0.026–0.085 cm^3^/g) (Table S1). In the absence of templates during the polymerization and cross-linking processes, the NIHs showed small SSAs (9.9–28.7 m^2^/g) as a result of large grain sizes and significant aggregation.

Suspended particle sizes of MIHs and NIHs were determined in methanol. The sizes of St-based, MAA-based, and MMA-based MIHs were 122–142, 122–164, and 190–255 nm, respectively. The particle sizes of NIHs, regardless of the types of functional monomers, were 255 nm (Figure 2f). These measurements were further confirmed by TEM analysis (Figure 2g,h). St-based MIH with BPA imprints and its corresponding NIH particles exhibited sizes of ca. 120 and 250 nm, respectively, which were close to those measured by DLS. The smaller particle sizes in the MIHs, compared to the NIHs, are attributed to hindered polymerization caused by the complexation of monomers with templates, resulting in shorter chains.^30^ The steric hindrance imposed by templates also led to a looser, nanoporous structure of St-based MIH particles, while St-based NIH particles were denser (Scheme 1). Based on the dependence of particle sizes on monomer–template interactions, the smaller particle sizes of St-based and MAA-based MIHs relative to those of MMA-based MIH suggest that the interactions between bisphenols and either St or MAA were stronger than those involving MMA. Particle aggregation of both MIHs and NIHs particles under dry conditions primarily arose from electrostatic attraction between SiO_2_ moieties on the surface. Since SiO_2_ clusters were formed through sol–gel reactions in the second stage to cross-link the preformed polymers during the preparation, a larger portion of SiO_2_ moieties was driven to be located in the outer region of hybrid particles.^7^ The uneven SiO_2_ distribution was more pronounced in the NIHs due to stronger interactions between the longer polymer chains formed in the absence of template-induced hindrance (Scheme 1b). Likewise, the more significant aggregation of BPA-imprinted hybrids, compared to 2BPF- and 4BPF-imprinted particles, suggests that the monomers/polymers interacted more strongly with BPA molecules than with the other two analogues. This hypothesis was further supported by subsequent adsorption results.

### 3.2 Adsorptions of MIHs Prepared with Different Template/Functional Monomer Associations

**Figure 3:**
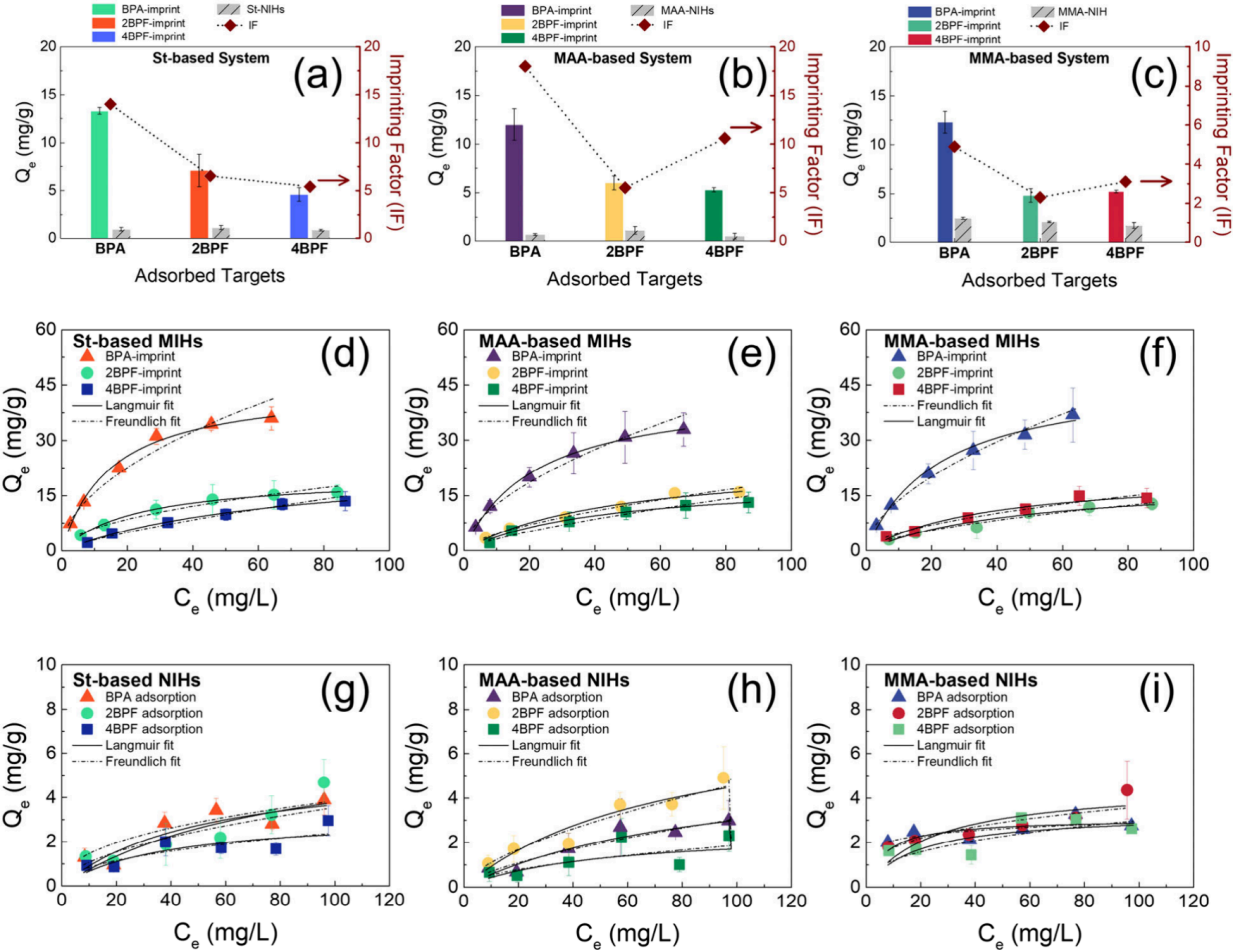
Adsorption ability and IFs of different imprints in (a) St-based, (b) MAA-based, and (c) MMA-based systems. Adsorption isotherms of (d) St-based, (e) MAA-based, and (f) MMA-based MIHs for their respective bisphenol templates and (g) St-based, (h) MAA-based, and (i) MMA-based NIHs for the three bisphenols at 298 K. *Q*_e_ and *C*_e_ are the adsorption amounts and concentrations of bisphenols at equilibrium, respectively.

The adsorption ability of MIHs prepared with different template/monomer associations and their corresponding NIHs was examined to evaluate their imprinting performance. For comparison, MIHs and NIH containing only MMA moieties were also prepared, and their adsorption abilities were measured. Figure 3a–c illustrates the adsorption ability of St-, MAA-, and MMA-based MIHs and NIHs for their respective templates at a constant concentration of 20 mg/L. Regardless of the functional monomers, MIHs exhibited similar adsorptions for their corresponding templates. BPA adsorptions (12.01–13.27 mg/g) were the highest, followed by 2BPF (4.88–7.06 mg/g) and 4BPF adsorptions (4.63–5.35 mg/g). In contrast to the high adsorptions by MIHs, NIHs exhibited low adsorptions for all three bisphenols (0.51–2.47 mg/g), confirming the imprinting effect. The imprinting factors (IFs) are calculated as the ratio of adsorption amounts (*Q*_e_) by MIHs to those of the corresponding NIHs at equilibrium (eq 2). Instead of adsorption capacity (*Q*_max_), *Q*_e_ values are used for IF calculation as they comprehensively account for imprinting efficiency and effectiveness (eq S1). Compared to 2BPF and 4BPF, -BPA template resulted in the highest IF values (14–18) in both the St- and MAA-based systems. While 4BPF imprinting had the lowest IF (5.4) in the St-based system, 2BPF imprinting led to the lowest IF (5.3) in the MAA-based system. Compared to the St- and MAA-based systems, the absence of primary functional monomers decreased the IF values (2.3–5.0) for all three bisphenol templates in the MMA-based system. Nevertheless, BPA imprinting still exhibited the highest IF value (5.0), while the lowest IF value was found with 2BPF (2.3).

Adsorption isotherms of MIHs for their corresponding bisphenol templates by at 298 K were further examined (Figure 3d–f) and fitted with the Langmuir (eq 6) and Freundlich models (eq 7) to determine the adsorption capacity and affinity.^31^
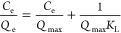
6
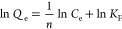
7where *C*_e_ (mg/L) is the bisphenol equilibrium concentration, *Q*_max_ (mg/g) is the calculated maximum adsorption capacity, *K*_L_ (L/mg) is the Langmuir binding affinity constant, and *K*_F_ and *n* are the Freundlich constants and adsorption favorability, respectively. Regardless of the functional monomers used, the BPA-imprinted MIHs demonstrated the highest adsorption capacity for BPA. Table 2 lists the adsorption capacity and parameters derived from the two model fittings. The isotherm parameters obtained from the St-based and MAA-based MIHs exhibited strong correlations with the Langmuir model (*R*^2^ > 0.961), while the adsorption isotherms of MMA-based MIHs were closer to the Freundlich model (*R*^2^ > 0.971). This finding, coupled with the higher IF values in the St- and MAA-based systems than in the MMA-based system, indicates that the St and MAA monomers enhance imprinting quality, thereby promoting specific interactions with these bisphenols. While the adsorptions of BPA by both the St- and MAA-based MIHs were better fitted with the Langmuir model, the adsorptions of 4BPF and 2BPF in the St-based and MAA-based systems, respectively, were slightly closer to those of the Freundlich model. This result correlated with the high IF values of BPA imprints (14–18) and relatively low IF values of the 4BPF imprints (5.4) and 2BPF imprints (5.3) in the St-based and MAA-based systems, respectively, suggesting superior structural directing ability of BPA with functional monomers to create the high quality of imprinted sites. On the other hand, π–π interactions of 4BPF molecules with St monomers and H-bond interactions of 2BPF molecules with MAA monomers are unfavorable.

**Table 2:** Isotherm Parameters of Different Functional Monomer-Based MIHs for Their Respective Bisphenol Templates

		Langmuir			Freundlich		
MIH	template	*Q*_max_ (mg/g)	*K*_L_ (×10^–2^ L/mg)	*R*^2^	*n*	*K*_f_	*R*^2^
St-based	BPA	44.8	6.74	0.994	1.94	4.83	0.977
	2BPF	20.4	4.35	0.998	1.97	1.87	0.978
	4BPF	26.7	1.22	0.962	1.33	0.52	0.981
MAA-based	BPA	43.7	4.61	0.998	1.77	3.43	0.981
	2BPF	24.9	2.14	0.961	1.60	1.08	0.990
	4BPF	19.6	2.34	0.984	1.91	0.59	0.939
MMA-based	BPA	47.6	4.52	0.986	1.79	3.81	0.994
	2BPF	19.6	2.02	0.872	1.71	0.95	0.971
	4BPF	21.0	2.69	0.933	1.82	1.34	0.974

Benefiting from effective imprinting with BPA templates, the BPA-imprinted hybrids exhibited the highest adsorption capacity (*Q*_max_ = 43.7–47.6 mg/g) and binding affinity (*K*_L_ = 4.52–6.74 L/mg) toward BPA compared to the 2BPF- and 4BPF-imprinted hybrids for their respective templates (*Q*_max_ = 19.6–26.7 mg/g, and *K*_L_ = 1.22–4.35 L/mg). The variations in adsorption capacities among different templates, yet similar adsorption capacities of a given compound across different functional monomer-based systems, reveal that the template conformation, rather than the type of functional monomer, primarily determines the imprinting efficiency. Although functional monomers have a weaker influence on imprinting efficiency, they significantly affect adsorption affinity. The highest affinity of BPA imprints for BPA molecules was observed in hybrids with St moieties (*K*_L_ = 6.74 L/mg), compared to those with MAA (*K*_L_ = 4.61 L/mg) and MMA moieties (*K*_L_ = 4.52 L/mg). Since H-bonds are stronger than π–π interactions,^14−16^ the advantage of St functional monomers in imprinting and rebinding is attributed to their hydrophobicity, which reduces solvent (ethanol or water) interference in template/monomer complexation and guest–host interactions. A similar trend was observed in 2BPF adsorption, but an opposite relationship occurred in 4BPF adsorption. While the lowest affinity of the 2BPF imprints was found in the MAA- and MMA-based systems (*K*_L_ = 2.07–2.14 L/mg), the weakest interaction between the 4BPF imprints and their template occurred in the St-based system (*K*_L_ = 1.22 L/mg). These results again correlate with the IF values, further supporting the unfavorable complexation between St monomers and 4BPF molecules. Additionally, H-bond formation with MAA moieties is more challenging for 2BPF, which has two *o*-OH groups. In contrast to the MIHs, NIHs in general exhibited low and nonspecific adsorptions (Figure 3g–i and Table S2).

### 3.3 Thermodynamic Parameters

**Figure 4:**
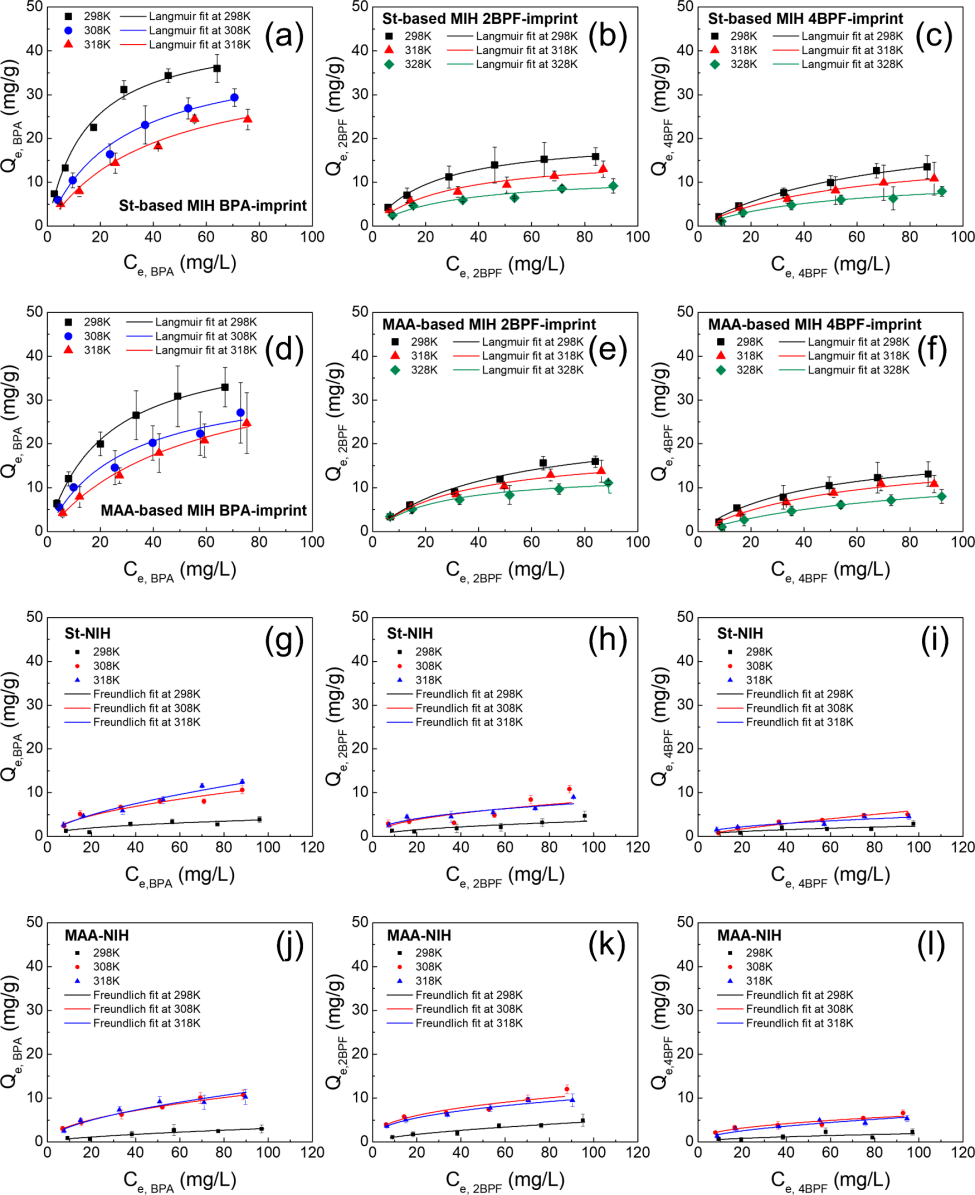
Adsorption isotherms of St-based MIHs with (a) BPA, (b) 2BPF, and (c) 4BPF imprints and those of MAA-based MIHs with (d) BPA, (e) 2BPF, and (f) 4BPF imprints for their respective templates at 298–328 K. Due to the similarity in the adsorption isotherms of 2BPF- and 4BPF-imprinted hybrids at 298 and 308 K, 328 K was selected to replace 308 K in these two systems to produce distinct adsorption results. Adsorption isotherms of St-based NIHs for (g) BPA, (h) 2BPF, and (i) 4BPF and MAA-based NIHs for (j) BPA, (k) 2BPF, and (l) 4BPF at 298–318 K. *Q*_e_ and *C*_e_ are the adsorption amounts and concentrations of bisphenols at equilibrium, respectively.

To further evaluate the effectiveness of imprinting from an energy perspective, we focused on St- and MAA-based systems to measure adsorption isotherms at different temperatures ranging from 298 to 328 K, as depicted in Figure 4. The adsorption capacities of the three bisphenols by both St- and MAA-based MIHs decreased with an increase in temperature (Figure 4a–f). In contrast, the adsorption ability of the corresponding NIHs increased with temperature (Figure 4g–l). Since NIHs lack customized binding sites, adsorption primarily occurred at external surfaces due to the weak driving force for bisphenol migration through the matrix. However, an increased thermal energy can facilitate molecular diffusion into the interior by overcoming internal resistance.^32,33^ Additionally, it weakens polymer chain interactions to promote bisphenol solubilization within the organic matrix, thus increasing the level of adsorption. Among the three bisphenols, 4BPF exhibited the lowest adsorption by NIHs, likely due to the rotational flexibility of its phenyl groups, which may hinder diffusion through the dense network. Despite the increase in adsorption with temperature, the isotherms of NIHs were poorly fitted by the Langmuir model (Tables S2–S4), supporting the idea that the adsorption is primarily governed by nonspecific hydrophobic interactions between bisphenol molecules and the organic components of the NIHs. In contrast, the customized binding sites in MIHs, confined by SiO_2_ moieties, still maintained Langmuir-type adsorption behavior, although their adsorption efficiency decreased at higher temperatures.

The Langmuir constants of MIHs at various temperatures (*K*_L–T_) were obtained from the Langmuir model fitting. Thermodynamic parameters, including Δ*G*_ads_^°^, Δ*H*_ads_^°^, and Δ*S*_ads_^°^, were then calculated based on the Langmuir constants using the van’t Hoff equation (eq 8) and Gibbs free energy of adsorption (eq 9),^34^ as listed in Table 3.
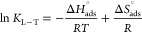
8
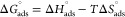
9where *K*_L–T_ (L/mol) is the Langmuir constant derived from various temperatures, *R* is the universal gas constant (8.314 J K^–1^ mol^–1^), and *T* (K) is the adsorption temperature. Negative Δ*G*_ads_^°^ values were obtained from all of the systems, indicating spontaneous adsorptions by the MIHs. Unlike 2BPF and 4BPF imprints, BPA imprints showed exclusively thermodynamically favorable adsorption in both St- and MAA-based systems for their template molecules. The highly negative Δ*H*_ads_^°^ values (−35.2 to −38.9 kJ/mol) for BPA adsorption indicate stable BPA/imprint complexes. Furthermore, the highly negative Δ*S*_ads_^°^ values (−40.5 to −50.6 J mol^–1^ K^–1^), as a result of the loss of randomness degree upon BPA adsorption, suggest intimate guest–host matching within the imprinted sites. Entropy not only relates to the ability of molecules to move, rotate, and vibrate within a confined space but also correlates with the extent of molecular substitution during adsorption.^35−37^ Compared to BPA imprints in MAA-based MIH (Δ*S*_ads_^°^ = −40.5 J mol^–1^ K^–1^), the more negative Δ*S*_ads_^°^ value (−50.6 J mol^–1^ K^–1^) was observed in the St-based MIH, indicating fewer pre-adsorbed water molecules were replaced per adsorption event. This supports the idea that the hydrophobic functional monomer enhances the imprinting quality by reducing the interference of the hydrophilic solvent in template/monomer complexation, leading to a well-defined imprint geometry for the template. Additionally, it facilitates BPA rebinding to the imprinted cavities by minimizing hindrance from pre-adsorbed water and/or promoted hydrophobic interactions. The higher degree of guest–host matching with St moieties is further supported by the higher adsorption heat released in the St-based MIH (Δ*H*_ads_^°^ = −38.9 kJ/mol) compared to the MAA-based system (Δ*H*_ads_^°^ = −35.2 kJ/mol).

**Table 3:** Thermodynamic Parameters of MIHs Prepared with Different Functional Monomers for Their Respective Bisphenol Templates

MIH	template	*T* (K)	Δ*G*_ads_^°^ (kJ/mol)	Δ*H*_ads_^°^ (kJ/mol)	Δ*S*_ads_^°^ (J mol^–1^ K^–1^)
St-based	BPA	298	–23.8	–38.9	–50.6
		308	–23.3		
		318	–22.8		
	2BPF	298	–22.4	–6.4	53.9
		318	–23.5		
		328	–24.1		
	4BPF	298	–19.7	14.1	112.2
		318	–21.7		
		328	–22.6		
MAA-based	BPA	298	–23.1	–35.2	–40.5
		308	–22.7		
		318	–22.3		
	2BPF	298	–20.6	16.1	123.1
		318	–23.1		
		328	–24.3		
	4BPF	298	–21.0	–15.9	17.0
		318	–21.4		
		328	–21.5		

In contrast to BPA adsorption, 2BPF and 4BPF adsorptions by their corresponding MIHs took place with a weak driving force (Δ*H*_ads_^°^ = −6.4 to −15.9 kJ/mol) or even required extra energy (Δ*H*_ads_^°^ = 8.9–16.1 kJ/mol) to proceed. The positive Δ*S*_ads_^°^ values of 2BPF and 4BPF adsorptions by their corresponding St- and MAA-based MIHs indicate replacement of multiple pre-adsorbed water molecules with one 2BPF or 4BPF adsorption. This behavior suggests weak complexation between these bisphenols with St or MAA monomers, leading to a low imprinting efficiency and quality. The hindered template/monomer complexation issue was more pronounced in the 4BPF/St and 2BPF/MAA systems, comprehensively resulting in endothermic adsorptions coupled with highly positive Δ*S*_ads_^°^ values (112.2–123.1 J mol^–1^ K^–1^), low adsorption affinity (*K*_L_ = 1.22–2.14 L/mg), low IF values (5.3–5.5), and Freundlich-closed adsorption behavior. Since all three bisphenols possess a similar molecular structure, the high imprinting performance with BPA could be attributed to its strong structural directing ability owing to its two additional -CH_3_ groups on the bridged carbon between its two phenyl moieties and *p*-OH groups.

### 3.4 Selectivity of MIHs for Different Bisphenols

**Figure 5:**
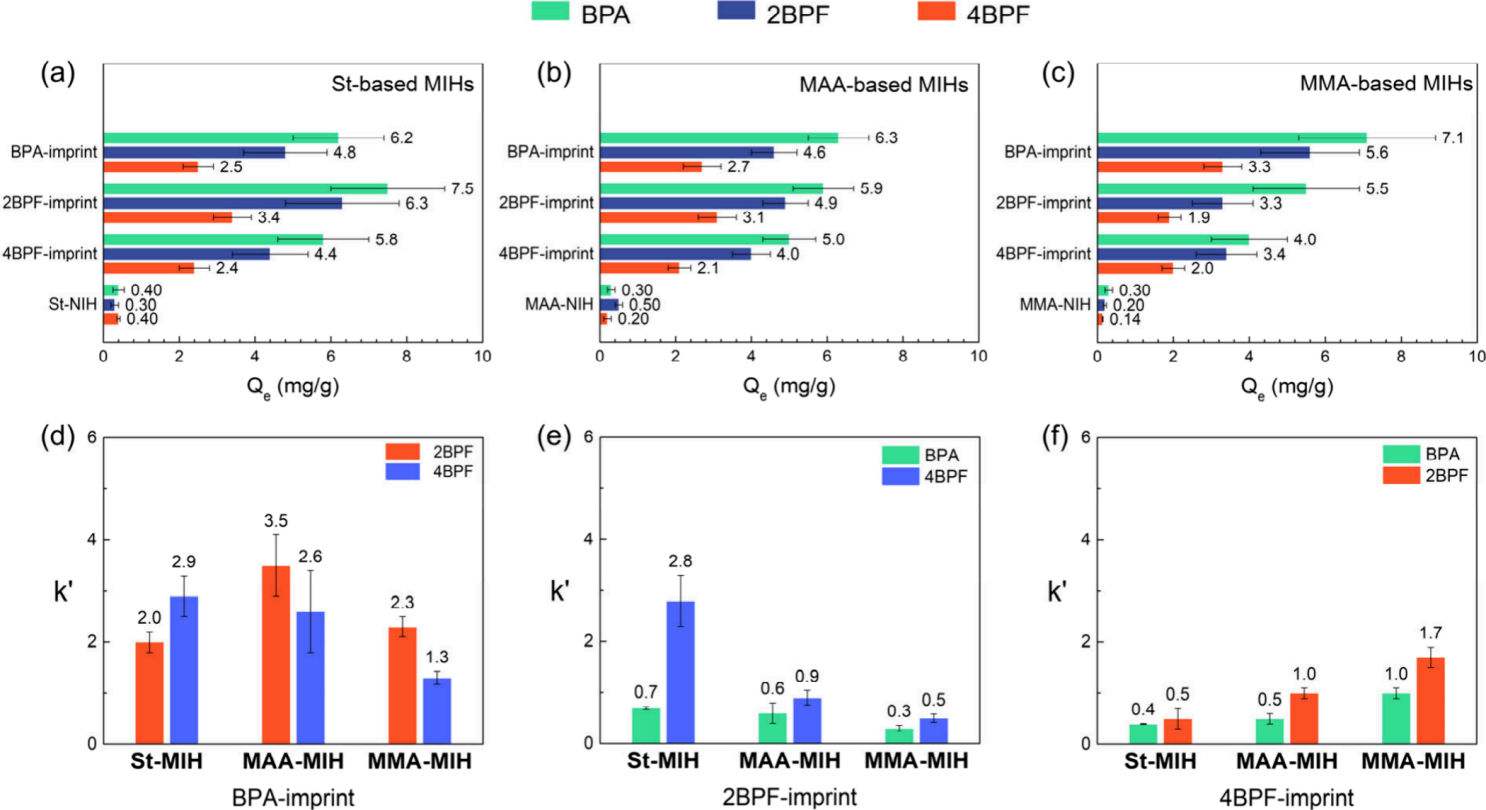
Competitive adsorption of bisphenols by (a) St-based, (b) MAA-based, and (c) MMA-based MIHs and corresponding NIHs in a mixture containing the three bisphenols, each at an initial concentration of 20 mg/L. Selectivity of (d) BPA, (e) 2BPF, and (f) 4BPF imprints of MIHs with different functional monomers.

Figure 5 illustrates the adsorption ability and selectivity of MIHs to the three bisphenols in the mixtures. The coexistence of structural analogues inhibited the adsorption of targets by their corresponding imprints. However, regardless of the types of imprints and functional monomers, BPA exhibited the highest competitive rebinding ability to the MIHs, with the order of binding ability being BPA > 2BPF > 4BPF (Figure 5a–c). Based on the adsorption behavior of bisphenols with different imprints, BPA has the potential to serve as a dummy template for 2BPF and 4BPF as BPA imprints exhibit comparable or even higher adsorption capacity for these analogues than their corresponding imprints. Similarly, 2BPF could act as a dummy template for BPA in the St-based system. The selectivity of MIHs for a templated target over analogues in the mixtures was evaluated based on the ratios of distribution coefficients between compounds (*K*_d_, eq 5). Since the apparent adsorption of MIHs comprised both nonspecific adsorption by the matrix and specific adsorption by the imprints, a relative selectivity coefficient (*k*′, eq 3), derived from the ratio of the selectivity coefficient (*k*, eq 4) of a MIH (*k*_MIH_) to that of its corresponding NIH (*k*_NIH_), was calculated. This coefficient accounts for nonspecific interactions and provides a more accurate evaluation of the selectivity of the imprints. If *k*′ exceeds unity, this indicates that the imprints exhibit preferential binding toward the target compared to the analogues. The MIHs with BPA imprints all demonstrated high BPA selectivity (*k*_BPA/analogues_^′^ = 1.3–3.5) over 2BPF or 4BPF, and the selectivity varied with functional monomers. Analysis of variance indicates that the selectivity variations among functional monomers were statistically significant (*p* = 0.0108–0.0262 (Table S5)). While BPA selectivities over 2BPF and 4BPF were 2.3 and 1.3, respectively, in the MMA-based MIHs, they increased to 3.5 and 2.6, respectively, in the MAA-based MIHs (Figure 5d). This reveals that H-bonds with MAA moieties can effectively distinguish BPA, which has two *p*-OH groups, from 2BPF, which contains two *o*-OH groups. Moreover, the two -CH_3_ groups on the bridged carbon atom of BPA synergistically promote preferential BPA adsorption relative to 4BPF to the imprinted sites. The BPA selectivity over 4BPF further increased to 2.9 in the St-based MIHs. Compared to the statistical difference between the MAA- and MMA-based systems (*p* = 0.0611), this selectivity in the St-based system differs more significantly from that in the MMA-based system, with a *p* value of 0.0169 (Table S6), indicating that St moieties are more effective than MAA moieties in distinguishing BPA from 4BPF. In contrast to the high BPA-to-4BPF selectivity (2.9), BPA-to-2BPF selectivity decreased to 2.0. As the distinct molecular feature of 4BPF compared to BPA and 2BPF is the rotational flexibility of its two phenyl groups, the high degree of exclusion of 4BPF from the BPA imprints is attributed to hindered π–π interactions with the St moieties.^38^ Moreover, a stabilized molecular conformation is the key factor governing selectivity in the St-based system.

In contrast to BPA imprints, 2BPF and 4BPF imprints showed less selectivity toward their template compounds compared to BPA (*k*_2BPF/BPA_^′^ = 0.3–0.7, and *k*_4BPF/BPA_^′^ = 0.4–1.0) (Figure 5e,f). This finding suggests that the two -CH_3_ groups, two *p*-OH groups, and restricted molecular conformation of BPA not only benefit molecular imprinting but also facilitate its rebinding to the nanocavities. Despite the low target-to-BPA selectivity, the 2BPF imprints with St moieties were preferentially bound with 2BPF over 4BPF (*k*_2BPF/4BPF_^′^ = 2.8), while the 4BPF imprints in MMA-based MIHs bound the template molecule more competitively than 2BPF (*k*_4BPF/2BPF_^′^ = 1.7). These phenomena again support the unfavorable formation of H-bond and π–π interactions with 2BPF and 4BPF, respectively.

### 3.5 Intermolecular Interactions

#### 3.5.1 Template/Monomer Complex

**Figure 6:**
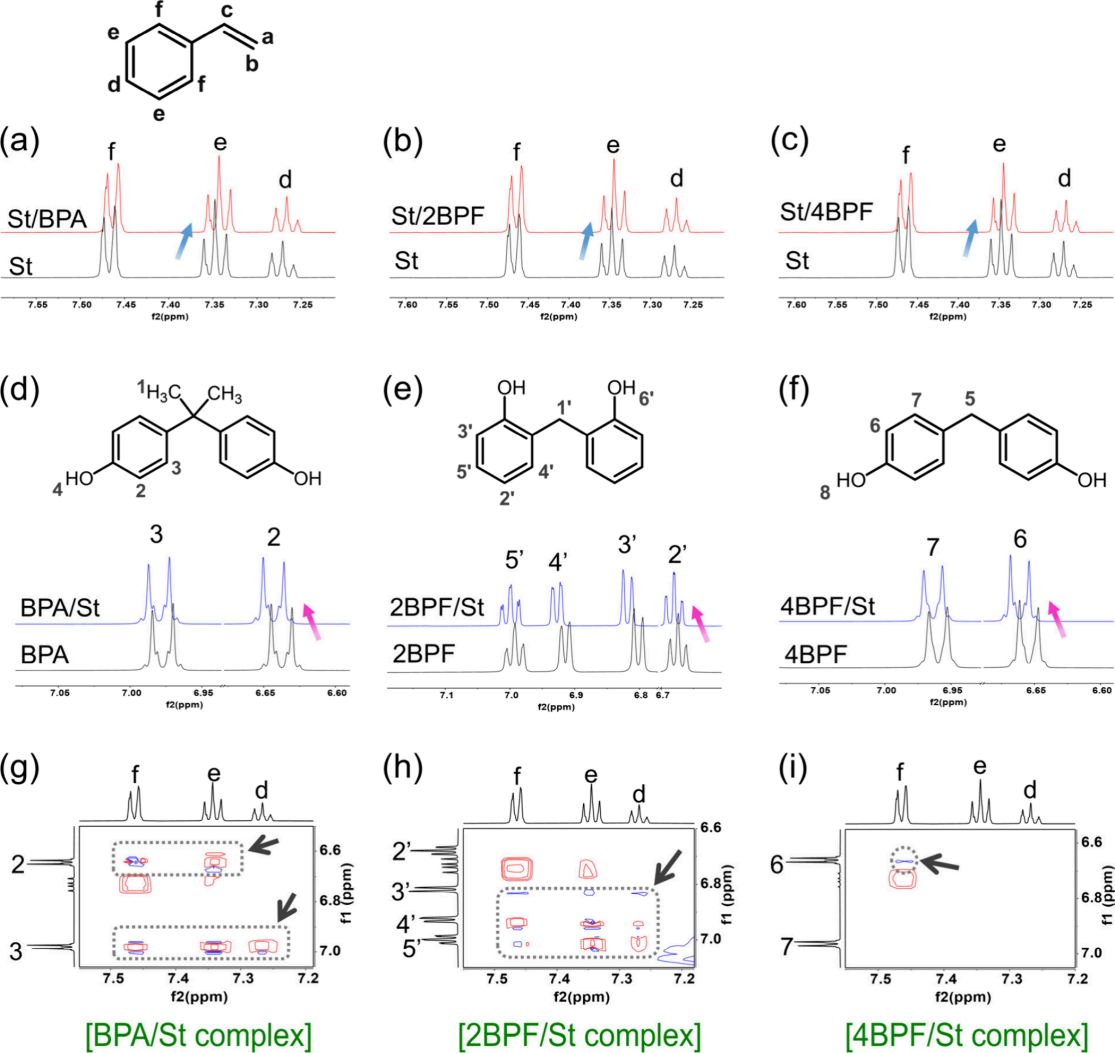
Partial ^1^H NMR spectra of (a–c) aromatic protons of styrene upon formation of complexes with bisphenols and aromatic protons of (d) BPA, (e) 2BPF, and (f) 4BPF upon formation of complexes with styrene. Partial ^1^H–^1^H NOESY NMR spectra of (g) BPA/St, (h) 2BPF/St, and (i) 4BPF/St complexes. The black arrows indicate aromatic protons of bisphenol/St NOE cross-peaks. The molar ratios of the bisphenol/St complexes were held at 1:2 in DMSO-*d*_6_. Full ^1^H NMR and ^1^H–^1^H NOESY NMR spectra are shown in Figures S4 and S5.

To determine whether the unique molecular configuration of BPA contributes to its superior structure-directing ability in imprinting and to ascertain the limitations of 2BPF and 4BPF in complexation with MAA/MMA and St monomers, respectively, intermolecular interactions between the functional monomers and the three bisphenols were analyzed using ^1^H NMR in DMSO-*d*_6_. Figure 6 illustrates the partial ^1^H NMR spectra of St, bisphenols, and the bisphenol/St mixtures with a controlled bisphenol:St molar ratio of 1:2. Upon complexation between St and bisphenols, upfield shifts of aromatic protons Hd–Hf of St were observed (Figure 6a–c), while the aromatic protons of all three bisphenols displayed downfield shifts (Figure 6d–f). The downfield and upfield shifts, resulting from deshielding and shielding effects, respectively, suggest aromatic donor–acceptor π–π interactions between St and the bisphenol molecules.^39^ The changes in chemical shifts (Δδ) of the aromatic protons of St, when associated with the three bisphenols individually, were recorded and are presented in Figure S3a. The largest upfield shifts in the BPA/St complex reveal the strongest interaction between the functional monomer and BPA, attributed to the presence of more electron-donating substituents on BPA (-CH_2_-(CH_3_)_2_) and more compatible π–π stacking conformations between the two molecules. Various π–π interaction geometries, such as face-to-face, parallel displaced, and edge-to-face arrangements, significantly influence the binding strength.^40^ To further identify the π–π stacking geometry, ^1^H–^1^H NOESY NMR analysis in DMSO-*d*_6_ was conducted for the three bisphenol/St complexes. As shown in Figure 6g, strong NOE cross-peaks were observed between the aromatic protons of St (Hd–Hf) and H3 of BPA, while H2 of BPA only correlated to He and Hf of St. This suggests a spatially close contact between the BPA and St (<4 Å) through face-to-face and/or parallel displaced π–π stacking interactions. For the 2BPF/St complex (Figure 6h), NOE cross-peaks between Hd–Hf of St and H4′ and H5′ of 2BPF, along with the chemical exchange-induced cross-peaks between H3′ of 2BPF and the aromatic protons of St, were observed. This suggests that the *o*-OH groups of 2BPF direct its phenyl groups to lean toward and contact the aromatic group of St. In contrast to BPA and 2BPF, only weak chemical exchange cross-peaks of H6 of 4BPF were found to correlate to Hf of St in the 4BPF/St complex (Figure 6i), suggesting an edge-to-face arrangement. The dissimilarity in bisphenol/St complexation geometries is associated with the chemical configuration of bisphenol molecules. The two bulky -CH_3_ groups on the bridged C atom restrict the rotation of the two phenyl groups in the BPA compound, thus facilitating the face-to-face complexation with styrene.^38^ Although 2BPF lacks the two -CH_3_ groups, its two adjacent *o*-OH groups still restrain the rotation of the two phenyl groups to a certain extent via an intramolecular H-bond to enable the π–π stacking.^41^ Unlike BPA and 2BPF, rotation of the phenyl groups is more profound in 4BPF due to absence of steric hindrance or intramolecular bonding.^38^ As a result, 4BPF exhibits the weakest imprinting ability in the St-based system.

**Figure 7:**
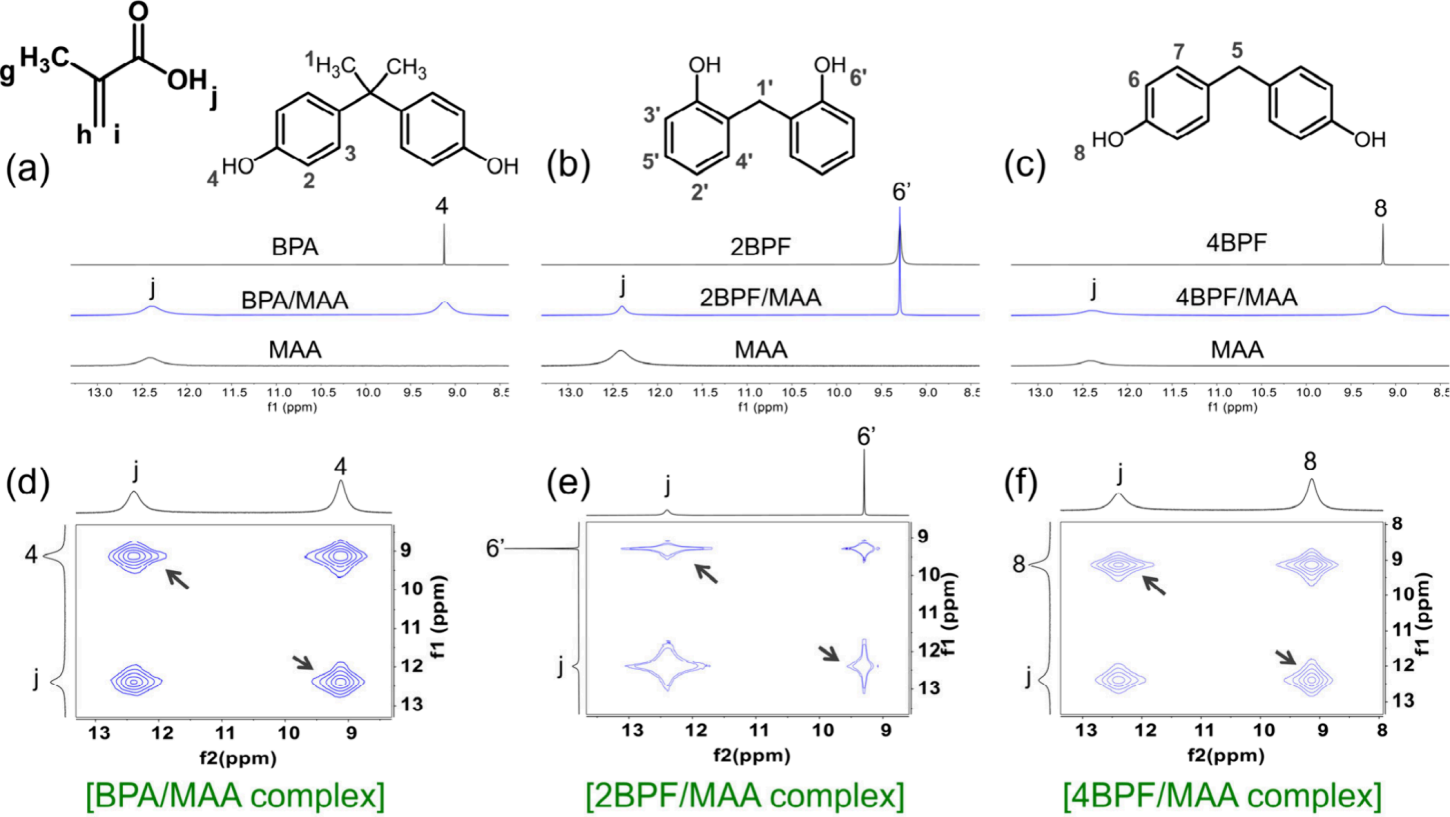
Partial ^1^H NMR spectra of the (a) BPA/MAA, (b) 2BPF/MAA, and (c) 4BPF/MAA complexes. Partial ^1^H–^1^H NOESY NMR spectra of the (d) BPA/MAA, (e) 2BPF/MAA, and (f) 4BPF/MAA complexes. The black arrows indicate the chemical exchange cross-peaks at -OH protons of bisphenol/MAA complexes. The molar ratios of the bisphenol/MAA complexes were held at 1:2 in DMSO-*d*_6_. Full ^1^H NMR and ^1^H–^1^H NOESY NMR spectra are shown in Figures S6 and S7.

MAA is expected to interact with the three bisphenols through hydrogen bonds via its -COOH groups. To confirm this interaction, the ^1^H NMR spectra of MAA and the MAA complexed with the three bisphenols individually were acquired, as shown in Figure 7. It was observed that the -OH proton peak of BPA or 4BPF was broadened and diminished when interacting with MAA as a result of fast proton exchange between the -OH groups and the -COOH groups of MAA (Figure 7a,c).^42^ However, such a phenomenon was less prominent in the MAA/2BPF complex (Figure 7b). The -OH protons of MAA showed the largest Δδ value when complexing with BPA, while the shift was the smallest when sociating with 2BPF (Figure S3b). Correspondingly, stronger chemical exchange cross-peaks between MAA and BPA or 4BPF compared to the MAA/2BPF complex were observed in the ^1^H–^1^H NOESY NMR spectra (Figure 7d–f). These findings indicate that the interaction of MAA with the two most distant *p*-OH groups of BPA and 4BPF molecules is more favorable than the interaction with the *o*-OH groups in 2BPF due to weaker steric effects and minimized charge repulsion.^41^ The electrostatic attraction between MAA and BPA and 4BPF stabilizes their complexes, thus improving the imprinting quality of these two bisphenols. On the other hand, the hindered interaction between MAA and 2BPF during imprinting not only results in low imprinting quality but also leads to the endothermic adsorption of 2BPF by its imprints in the MAA-based system.

**Figure 8:**
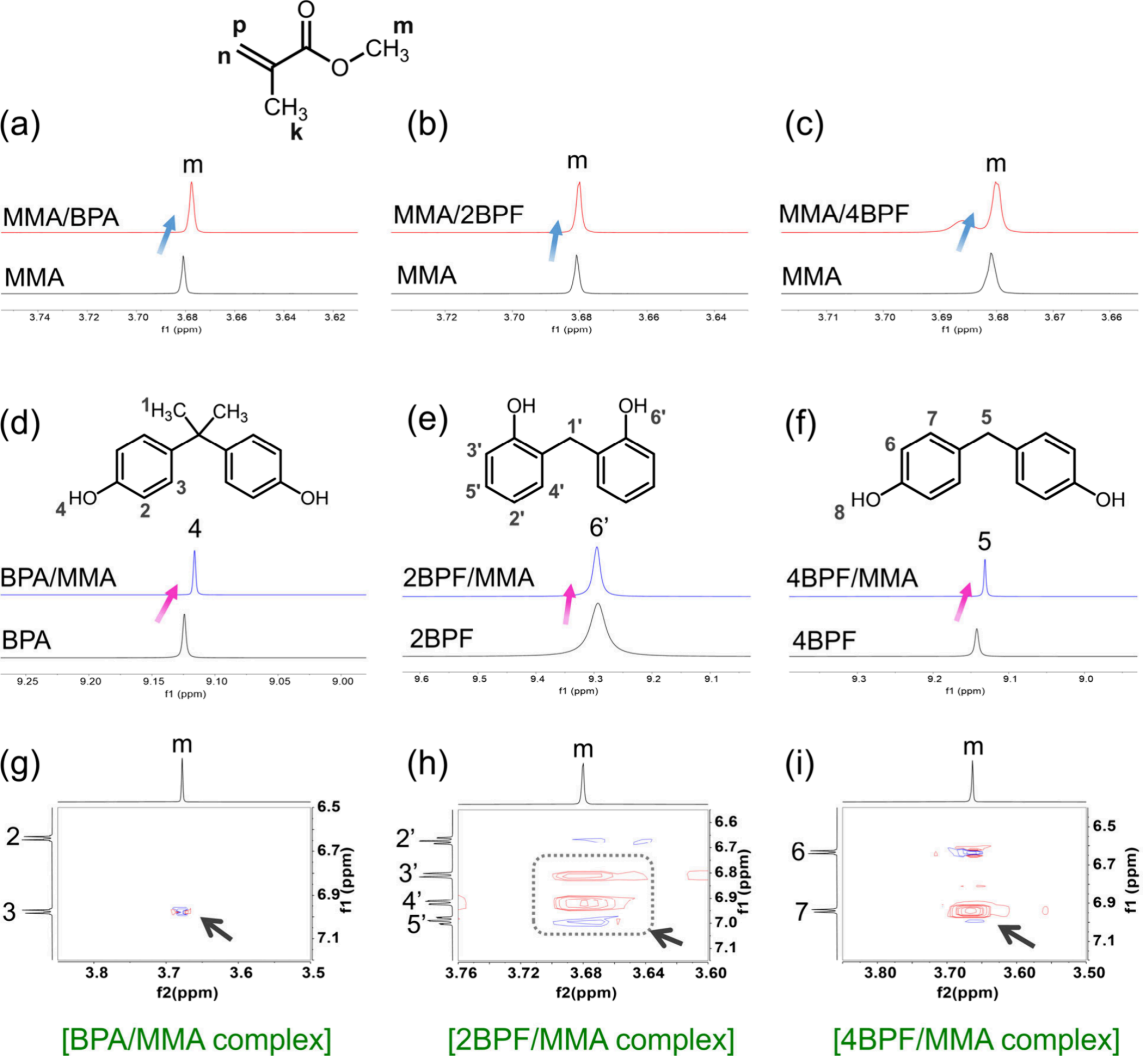
Partial ^1^H NMR spectra of (a–c) -OCH_3_ protons of MMA upon formation of complexes with bisphenols and -OH protons of (d) BPA, (e) 2BPF, (f) 4BPF upon formation of complexes with MMA. Partial ^1^H–^1^H NOESY NMR spectra of (g) BPA/MMA, (h) 2BPF/MMA, and (i) 4BPF/MMA complexes. The black arrows indicate bisphenol/MMA NOE cross-peaks. The molar ratios of bisphenol/MMA complexes were held at 1:2 in DMSO-*d*_6_. Full ^1^H NMR and ^1^H–^1^H NOESY NMR spectra are shown in Figures S8 and S9.

**Scheme 2:**
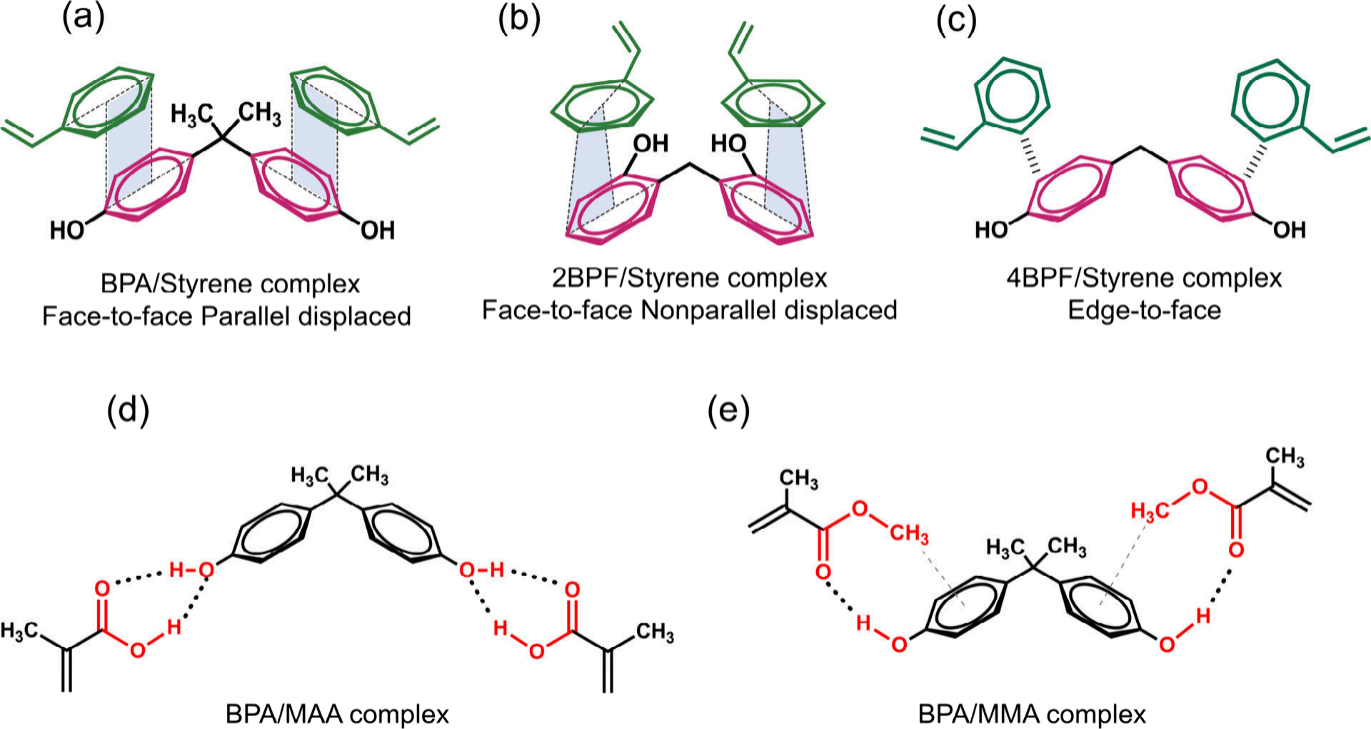
Proposed Geometries of π–π Interactions between St and Bisphenols in (a) BPA/St, (b) 2BPF/St, and (c) 4BPF/St Complexes and H-Bond and CH−π Interactions of BPA with (d) MAA and (e) MMA Monomers

MMA molecules were found to interact with the three bisphenols via CH−π and H-bond interactions with their -OCH_3_ (Figure 8a–c) and C=O groups (Figure 8d–f), respectively, as shown in Figure 8. The CH−π interaction mainly occurred between the -OCH_3_ groups of MMA and the aromatic rings of the bisphenols (Figure 8g–i). Relative to the interactions with 2BPF and 4BPF, the CH−π interaction was stronger in the BPA/MMA complexes according to the large Δδ value of the -OCH_3_ protons (Figure 8a–c and Figure S3c). The polar attraction between the -OH groups of the bisphenols and the C=O group of MMA shifted the -OH proton peak to an upfield region (Figure 8d–f). Similar to the H-bond interactions with MAA molecules, BPA and 4BPF exhibited stronger H-bond interactions with MMA but not in the case of 2BPF. The H-bond and CH−π interactions stabilize the BPA/MMA complexes more effectively compared to 2BPF/MMA and 4BPF/MMA complexes, thus resulting in the highest IF for this template. However, despite these interactions, the low Langmuir-correlated adsorption of MMA-based MIHs (Table 2) indicates that the noncovalent interactions involving only MMA monomers are not as strong as the π–π and H-bond interactions with St and MAA monomers, respectively, in creating specific binding sites. Scheme 2 illustrates the different interactions between bisphenols and the monomers. Overall, the strong capability of BPA in directing monomers to achieve efficient and effective imprinting is comprehensively due to its two -CH_3_ groups on the bridged C atom, restricted rotation of the two phenyl groups, and two *p*-OH groups that are far apart. This configuration enables BPA to form stable complexes with monomers through π–π stacking, H-bonds, or weak van der Waals forces. As a result, more templates can be incorporated into the polymeric matrix to increase the adsorption capacity of MIHs. Meanwhile, polymerization along the shape of the template creates imprinted cavities that fit better with the BPA molecules.

#### 3.5.2 Template/Polymer Interactions

**Figure 9:**
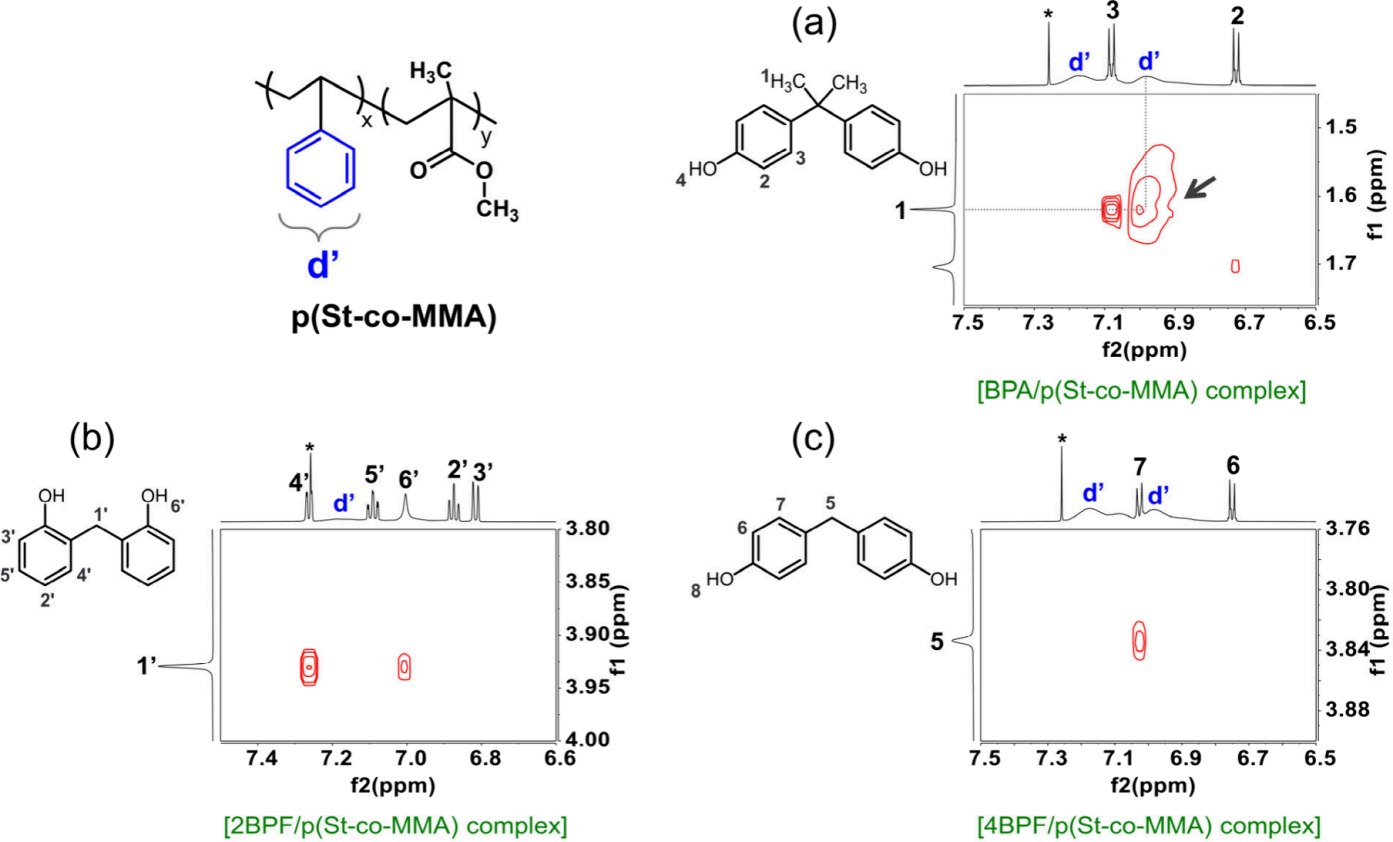
Partial ^1^H–^1^H NOESY NMR spectra of p(St-*co*-MMA) associated with (a) BPA (the black arrows indicate CH−π NOE cross-peaks), (b) 2BPF, and (c) 4BPF in CDCl_3_. Full ^1^H–^1^H NOESY NMR spectra are shown in Figure S11.

**Figure 10:**
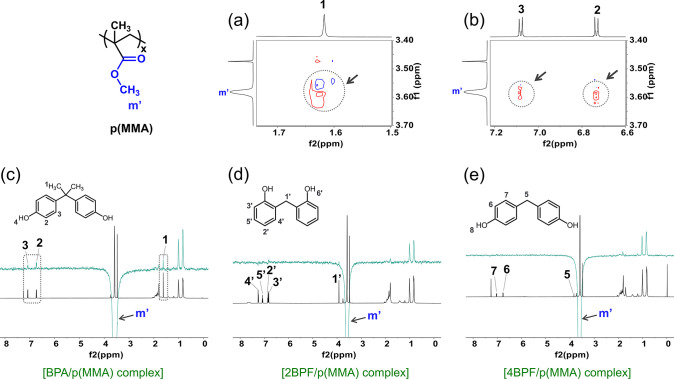
(a and b) Partial ^1^H–^1^H NOESY NMR spectra of p(MMA) upon association with BPA in CDCl_3_. ^1^H ROESY NMR spectra of p(MMA) upon association with (c) BPA, (d) 2BPF, and (e) 4BPF in CDCl_3_. The -OCH_3_ proton of p(MMA) was selectively irradiated (the downward peak shown in green spectra at ca. 3.6 ppm) to observe the intermolecular interaction with bisphenol templates. Full ^1^H–^1^H NOESY NMR spectra are shown in Figure S13.

As mentioned above, BPA demonstrated the strongest competitive rebinding ability to MIHs while coexisting with the other two analogues, regardless of whether 2BPF or 4BPF served as the template. The distinguishing feature of BPA compared to 2BPF and 4BPF may lie in its two -CH_3_ substituents on the bridged carbon atom between the two phenyl groups, which not only restrict the rotation of the phenyl groups but also interact with copolymers via van der Waals forces. To further elucidate the exceptional BPA adsorption attributed to the -CH_3_ groups, we analyzed the association of the three bisphenols with p(St-*co*-MMA), p(MAA-*co*-MMA), and pMMA using ^1^H–^1^H NOESY NMR in CDCl_3_ or DMSO-*d*_6_. As illustrated in Figure 9a, a strong NOE cross-peak was observed between the -CH_3_ protons of BPA and the aromatic protons of the phenyl group in p(St-*co*-MMA), indicating the proximity of the -CH_3_ groups to the aromatic rings. While such CH−π interaction was also evident in the St/BPA complexes (Figure S10), it appeared to be more pronounced when BPA interacted with the copolymer because of a weaker steric effect compared to the π–π interaction. The CH−π interaction is a significant noncovalent attractive force to drive aromatic–aliphatic interactions for molecular recognition.^43−45^ In this study, such a CH−π interaction could initially drive BPA to approach the imprinted site and then facilitate BPA to fit into the cavity. In contrast, no discernible NOE cross-peak was observed between the aromatic rings of the copolymer and the bridged -CH_2_ protons of 2BPF and 4BPF (Figure 9b,c). For interaction with the MAA-containing copolymer, the H-bond interaction between the -OH groups of BPA and the -COOH groups of the MAA moieties was still the dominant force that bound BPA to p(MAA-*co*-MMA) (Figure S12). In the absence of St or MAA moieties, the -CH_3_ groups and aromatic rings of BPA were found to interact with the -OCH_3_ groups of pMMA via a dispersion force and the CH−π interaction, respectively (Figure 10a,b). To validate these weak through-space interactions, ^1^H ROESY NMR analysis was further carried out owing to its enhanced sensitivity. The -OCH_3_ proton (ca. 3.6 ppm) of pMMA was selectively irradiated to observe its intermolecular interactions with the BPA molecules (Figure 10c). While the -OCH_3_ protons showed intermolecular interactions with the aromatic (ca. 6.7 and 7.1 ppm) and -CH_3_ protons (ca. 1.6 ppm) of BPA molecules, no observable ROE signals were detected for 2BPF and 4BPF molecules (Figure 10d,e).

**Scheme 3:**
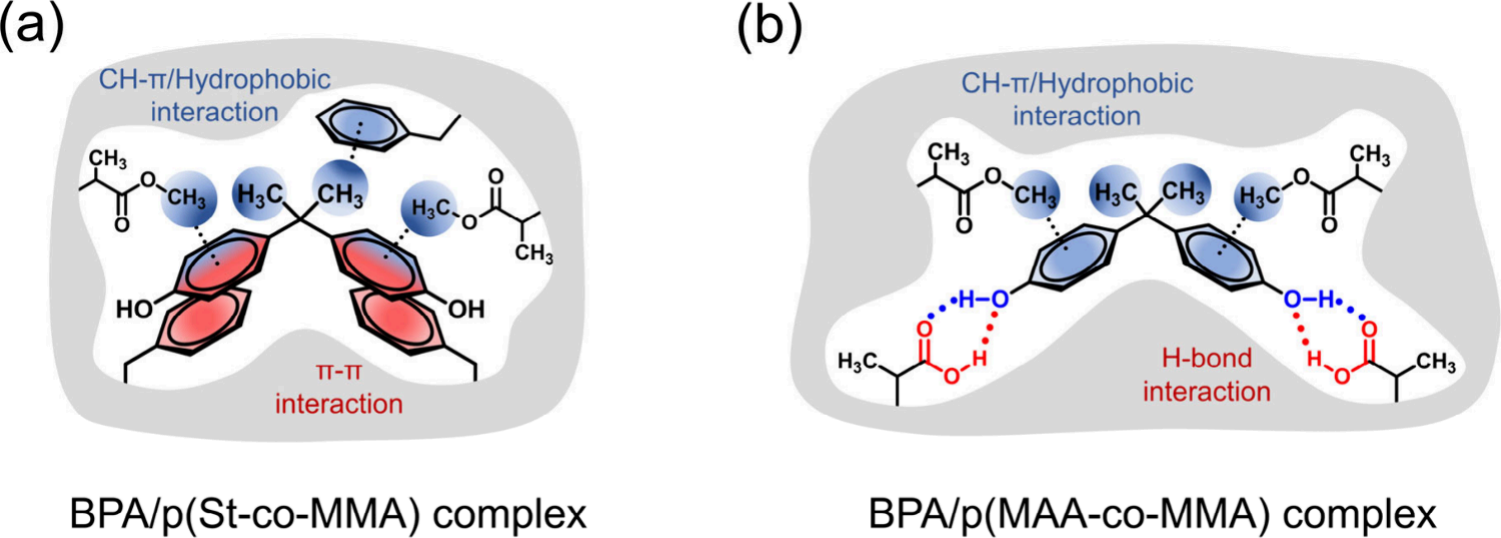
Proposed Intermolecular Interactions between BPA and (a) p(St-*co*-MMA) and (b) p(MAA-*co*-MMA)

Based on the NMR characterizations, the intermolecular interactions between BPA and copolymers are illustrated in Scheme 3. The higher affinity of BPA over 2BPF and 4BPF for the MIHs stems also from its additional -CH_3_ substituents on the bridged carbon atom, *p*-OH groups, and restricted molecular conformation. The critical role of the -CH_3_ groups in promoting and stabilizing the binding interactions of BPA has been demonstrated in many biological activities.^46,47^ In this study, the pair of -CH_3_ groups enables BPA to additionally interact with MMA moieties via van der Waals forces in all of the copolymers. Moreover, they restrict the rotation of the two phenyl groups in BPA, facilitating the CH−π interaction between the -OCH_3_ group of the MMA moieties and the aromatic ring of BPA molecules.^38^ These additional interactions promote the competitive adsorption of BPA in all of the imprinted systems even though the imprinted sites are not customized for this compound. In contrast, the weakest rebinding ability of 4BPF in the competitive systems is presumably due to its rotating phenyl groups, which hinder its interaction with MIHs.

## 4 Conclusions

The key molecular features of templates and functional monomers that govern the imprinting and rebinding ability of MIHs among three bisphenol analogues, BPA, 2BPF, and 4BPF, have been identified. Compared to hydrophilic MAA monomers, hydrophobic St monomers are more suitable for BPA imprinting as they inhibit the interference of a water/polar solvent during template/monomer complexation and target rebinding, thus enabling MIHs to exhibit high affinity for BPA. While functional monomers primarily influence the strength of binding of MIHs to the target, molecular configurations of templates comprehensively determine adsorption capacities, affinity, and selectivity. BPA has the strongest structural directing ability, compared to 2BPF and 4BPF, to achieve high imprinting efficiency and effectiveness for superior adsorption capacity and selectivity owing to its two additional -CH_3_ groups on the bridged carbon, *p*-OH groups, and restricted molecular conformation. These features also allow BPA to be more competitive in rebinding to the imprinted sites in the coexistence of the other two analogues even though the sites are created by structural analogues. In contrast, 4BPF is the least competitive to the binding sites, presumably due to its rotating phenyl groups. The rotational flexibility of 4BPF also limits its π–π interactions with St monomers, while the two *o*-OH groups of 2BPF hinder H-bond interactions with MAA monomers. The optimal template/functional monomer combinations for high adsorption performance include BPA/St, 2BPF/St, and 4BPF/MAA. Moreover, BPA has the potential to serve as a dummy template for 2BPF and 4BPF across the studied systems, while 2BPF can play a similar role for BPA in the St-based system.

## Supporting Information Available

Characterization details, including measurement of N_2_ adsorption–desorption isotherms, SEM, TEM, DLS, and LC-MS/MS; statistical analysis; calculation of imprinting factors; BET surface areas and pore volumes of the different MIHs and the corresponding NIHs; isotherm parameters of different functional monomer-based NIHs for bisphenols; supporting table for statistical analysis; SEM images of different functional monomer-based MIHs; N_2_ adsorption–desorption isotherm curves and pore size distributions; ^1^H NMR chemical shift changes (Δδ) of functional monomers after complexing with bisphenols; ^1^H NMR spectra of bisphenols and functional monomers before and after complexation; ^1^H–^1^H NOESY NMR spectra of bisphenol/functional monomer complexes; and ^1^H–^1^H NOESY NMR spectra of bisphenol/polymer complexes (PDF)
