# Supplementary material for: Insights into the Imprinting and Rebinding Performance of Molecularly Imprinted Hybrids for Bisphenol A and Bisphenol F
Source: ACS Appl Mater Interfaces. 2025 Apr 30;17(19):28568–84. doi: 10.1021/acsami.5c03038 (PMC12086839; doi:10.1021/acsami.5c03038)
Supplement: Supplementary file 1 — am5c03038_si_001.pdf [file am5c03038_si_001.pdf]

Supporting Information

Insights into the Imprinting and Rebinding Performance of Molecularly Imprinted Hybrids for Bisphenol A and Bisphenol F

Kae-Zheng Chin[a,b] *and Sue-min Chang*[a,]*

[a]Institute of Environmental Engineering, National Yang Ming Chiao Tung University, 1001, University Road, Hsinchu, 300093, Taiwan

[b]Graduate Institute of Environmental Engineering, National Taiwan University, No. 1, Section 4, Roosevelt Rd., Da'an District, Taipei 10617, Taiwan

*Corresponding Author:

E-mail: chang@nycu.edu.tw; Tel: +886-3-5712121 ext. 55506; Fax: +886-3-5725958

Characterizations

Surface morphologies of MIHs and NIHs were observed using a field emission scanning electron microscope (FE-SEM, Hitachi SU-8010) operated at an acceleration voltage of 15 kV. The sample powders were coated with Pt using a sputter coater for 90 s before analysis. Microstructure of St-based MIH for BPA-imprint and its corresponding St-based NIH were characterized by transmission electron microscope (TEM, JEOL, JEM-3000F) operated at an acceleration voltage of 200 kV. N_2 adsorption–desorption isotherms of the MIHs and NIHs were measured at 77 K using a gas sorption analyzer (Micromeritics, Tristar 3000). Prior to measurement, the sample powders were degassed at 60°C for 6 h under vacuum on a degas system (Micromeritics, VacPrep 061). Specific surface areas and pore size distributions were further derived from the adsorption data using the Brunauer-Emmett-Teller (BET) and the Barrett-Joyner-Halenda (BJH) models, respectively, and the total volume was determined from the amount of nitrogen adsorbed at relative pressure close to 1 ($P/P_0 = 0.99$). Hydrodynamic size distribution of MIHs and NIHs were analyzed using dynamic light scattering (DLS, Malvern, Nano ZS90). All the sample powders were ground before analysis to avoid aggregation. An adequate amount of powder was dispersed in methanol and sonicated for 5 min to assist the dispersion of particles. DLS measurement was taken at 25°C with a detector angle of 90°. Liquid chromatography-tandem mass spectrometry (LC-MS/MS, Agilent 6470) was employed to quantify the residue level of BPA in the extracted St-based MIHs. The BPA residue in St-based MIH was further extracted by washing the MIH powder with 10 mL of methanol/acetic acid (9:1 v/v%). The collected eluent was evaporated at 50°C under the flow of N_2 gas, and then the volume of the concentrated solution was adjusted by methanol to 1.0 mL before chromatography analysis.

Statistical Analysis

Single factor ANOVA was performed to identify the possibility of relative selectivity coefficient (k') for bisphenol analogs across the three different functional monomer systems were statistically significant. In addition, a t-test was used to determine the statistical difference of selectivity (k') between two functional monomers-based MIHs. The experimental data were repeated at least 3 times and expressed as mean (± standard deviation). All the statistical analysis was performed using Microsoft Excel 365 to calculate the *p*-value, where p-value < 0.05 was considered statistical significance.

Calculation of Imprinting Factors

In this study, the imprinting factors (IFs) are calculated as the ratios of equilibrium adsorption amounts (Q_e, mg/g) by MIHs to those by the corresponding NIHs at equilibrium. According to the Langmuir equation (**eq. S1**), Q_e is associated with both adsorption capacity (Q_{max}, mg/g) and Langmuir adsorption coefficient (K_L, L/mol), which represent imprinting efficiency and effectiveness, respectively. IFs calculated based on Q_e can provide a comprehensive evaluation for imprinting performance.

$$Q_e = Q_{max} \times \frac{K_L \times C_e}{1 + K_L \times C_e} \qquad \text{(S1)}$$

where C_e (mg/L) is the equilibrium concentration.

Table S1. BET surface areas and pore volume of the different MIHs and the corresponding NIHs.

Adsorbents	Templates	SSA[a] (m^2/g)	Pore Size (nm)	Pore Volume (cm^3/g)
St-based MIHs	BPA	10.2	3.6	0.033
	2BPF	73.2	3.6	0.064
	4BPF	51.8	3.8	0.085
MAA-based MIHs	BPA	10.1	3.6	0.011
	2BPF	64.7	3.9	0.040
	4BPF	27.8	3.7	0.026
MMA-based MIHs	BPA	16.6	3.8	0.013
	2BPF	99.0	3.9	0.042
	4BPF	79.0	3.9	0.037
St-based NIHs	-[b]	10.5	3.8	0.022
MAA-based NIHs	-	28.7	4.1	0.035
MMA-based NIHs	-	9.9	3.9	0.018

[a]SSA means the specific surface area. [b]– there were no templates involved.

Table S2. The isotherm parameters of NIHs for bisphenols analytes at 298K.

NIHs	Targets	Langmuir			Freundlich		
		Q_{max} (mg/g)	K_L ($\times 10^{-2}$ L/mg)	R^2	n	K_f	R^2
St-based	BPA	5.6	1.94	0.624	2.39	0.559	0.900
	2BPF	6.9	1.22	0.489	1.93	0.326	0.782
	4BPF	3.2	2.63	0.679	2.21	0.297	0.752
MAA-based	BPA	5.7	1.12	0.531	1.57	0.162	0.833
	2BPF	7.9	1.32	0.670	1.61	0.263	0.939
	4BPF	2.5	2.01	0.375	1.86	0.158	0.612
MMA-based	BPA	3.1	14.1	0.955	7.05	1.504	0.579
	2BPF	4.6	3.99	0.859	3.24	0.866	0.850
	4BPF	3.3	5.34	0.799	2.54	0.487	0.539

Table S3. The isotherm parameters of NIHs for bisphenols analytes at 308K.

NIHs	Targets	Langmuir			Freundlich		
		Q_{max} (mg/g)	K_L ($\times 10^{-2}$ L/mg)	R^2	n	K_f	R^2
St-based	BPA	13.1	3.25	0.932	1.89	0.983	0.920
	2BPF	15.3	1.35	0.321	2.15	0.958	0.672
	4BPF	10.2	1.11	0.874	1.29	0.173	0.955

	BPA	14.4	2.88	0.947	2.01	1.140	0.992
MAA-based	2BPF	13.4	4.09	0.874	2.70	1.988	0.927
	4BPF	7.9	3.16	0.799	2.46	0.927	0.886

Table S4. The isotherm parameters of NIHs for bisphenols analytes at 318K.

NIHs	Targets	Langmuir			Freundlich		
		Q_{max} (mg/g)	K_L ($\times 10^{-2}$ L/mg)	R^2	n	K_f	R^2
St-based	BPA	20.0	1.67	0.831	1.64	0.797	0.977
	2BPF	9.9	3.43	0.828	2.49	1.233	0.872
	4BPF	5.7	3.19	0.852	2.29	0.604	0.923
MAA-based	BPA	13.4	3.48	0.982	1.83	0.965	0.937
	2BPF	11.4	4.99	0.966	2.69	1.794	0.981
	4BPF	6.7	3.39	0.932	1.91	0.521	0.878

Table S5. *p*-values obtained by ANOVA for relative selectivity coefficient (k') of MIHs between the three different functional monomers.

Templated-MIHs	Bisphenol Analogs	k'			*p*-value
		St-MIH	MAA-MIH	MMA-MIH	
BPA-imprint	2BPF	2.0	3.5	2.3	0.0108
	4BPF	2.9	2.6	1.3	0.0262

2BPF-imprint	BPA	0.7	0.6	0.3	0.0063
	4BPF	2.8	0.9	0.5	0.0003
4BPF-imprint	BPA	0.4	0.5	1.0	0.0002
	2BPF	0.5	1.0	1.7	0.0003

Table S6. *p*-values of t-tests for the relative selectivity coefficient (k') of MIHs between two different functional monomers.

Imprint types	Target/Analogs	k'		
BPA-imprint	BPA/Analogs	St-MIH	MMA-MIH	*p*-value
	2BPF	2.0	2.3	0.2508
	4BPF	2.9	1.3	0.0169
		MAA-MIH	MMA-MIH	*p*-value
	2BPF	3.5	2.3	0.0701
	4BPF	2.6	1.3	0.0611
		St-MIH	MAA-MIH	*p*-value
	2BPF	2.0	3.5	0.0401
	4BPF	2.9	2.6	0.5602
2BPF-imprint	2BPF/Analogs	St-MIH	MMA-MIH	*p*-value
	BPA	0.7	0.3	0.0031
	4BPF	2.8	0.5	0.0169
		MAA-MIH	MMA-MIH	*p*-value
	BPA	0.6	0.3	0.0846
	4BPF	0.9	0.5	0.0152
		St-MIH	MAA-MIH	*p*-value

	BPA	0.7	0.6	0.2372
	4BPF	2.8	0.9	0.0211
	4BPF/Analogs	St-MIH	MMA-MIH	*p*-value
	BPA	0.4	1.0	0.0063
	2BPF	0.5	1.7	0.0012
		MAA-MIH	MMA-MIH	p-value
4BPF-imprint	BPA	0.5	1.0	0.0041
	2BPF	1.0	1.7	0.0055
		St-MIH	MAA-MIH	*p*-value
	BPA	0.4	0.5	0.2014
	2BPF	0.5	1.0	0.0202

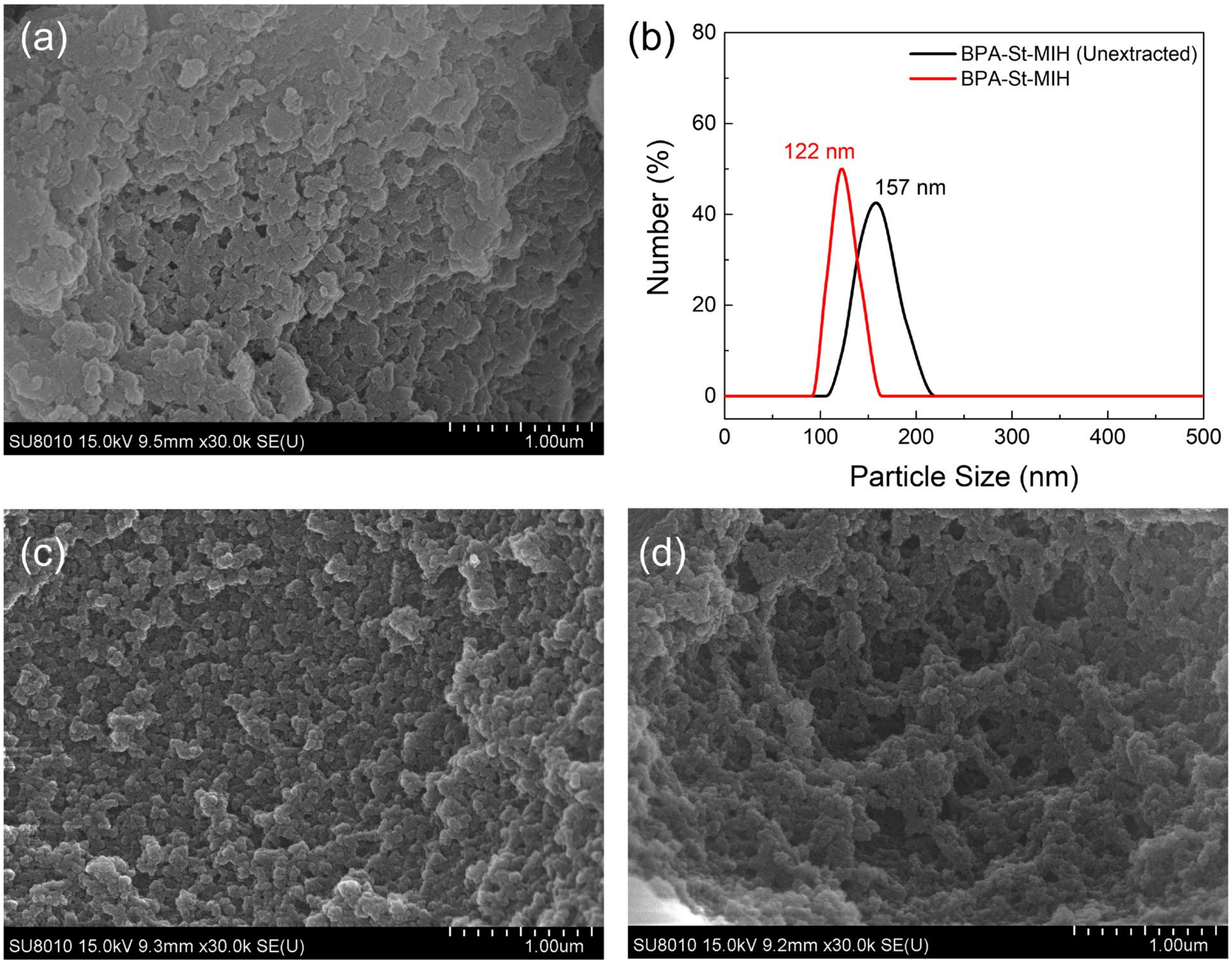

Figure S1. SEM images of (a) unextracted St-based MIH with BPA-imprint and the (b) particle size distribution of unextracted and extracted St-based MIH with BPA-imprints. SEM images of (c) MAA-based and (d) MMA-based MIH with BPA-imprint.

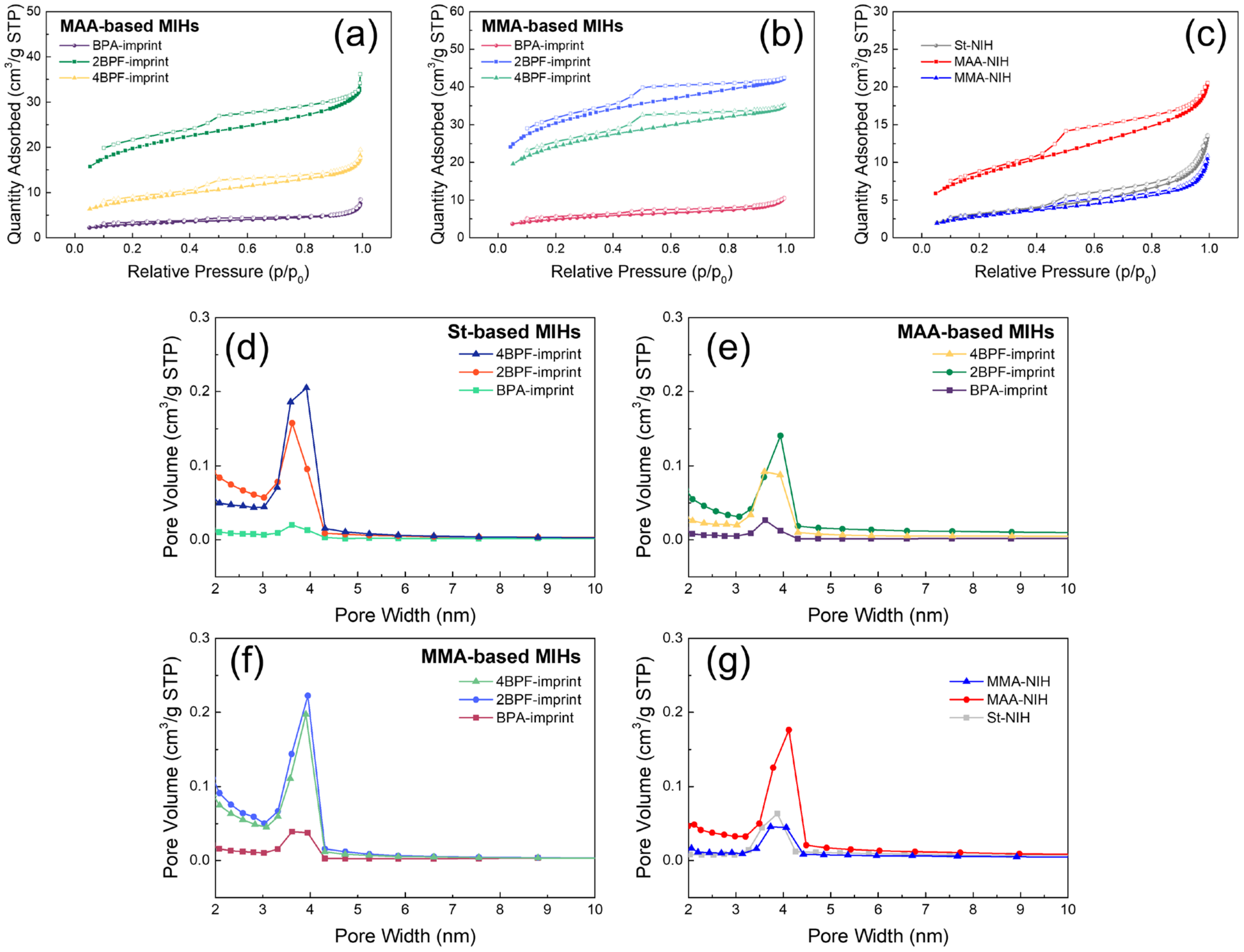

Figure S2. N_2 adsorption-desorption isotherm curves of (a) MAA-based MIHs, (b) MMA-based MIHs, and (c) NIHs. The solid symbol represents N_2 adsorption, and the open symbol represents N_2 desorption. Pore size distribution of (d) St-based MIHs, (e) MAA-based MIHs, (f) MMA-based MIHs, and (g) NIHs.

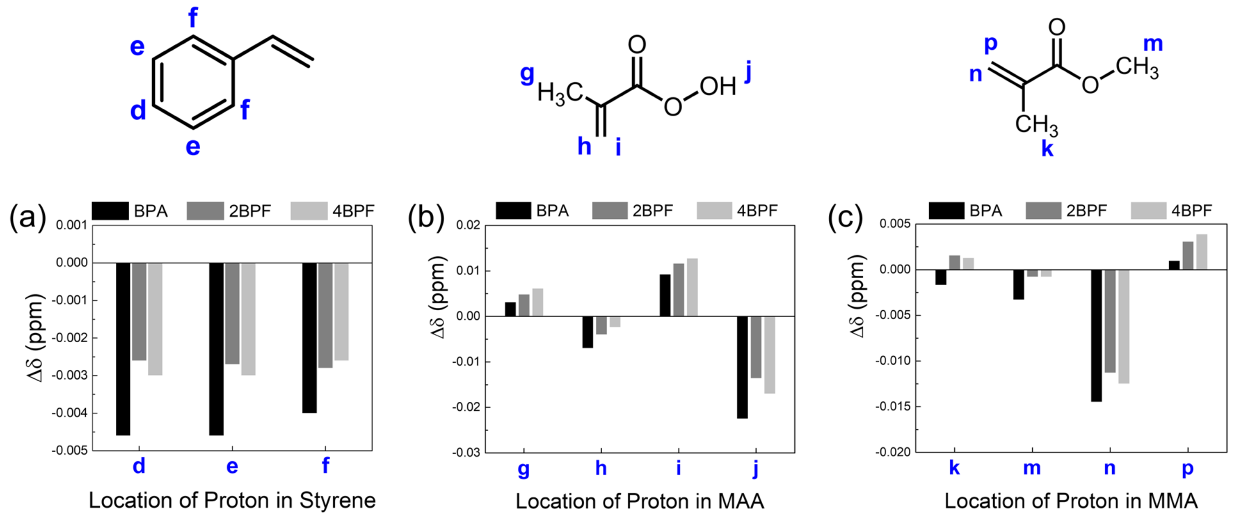

Figure S3. ^{1}H NMR chemical-shift changes (Δδ) of functional monomers: (a) St, (b) MAA, and (c) MMA upon association with bisphenols in DMSO-d_6. The molar ratio of bisphenol to functional monomer was controlled at 1:2. Δδ = δ (complex) – δ (individual).

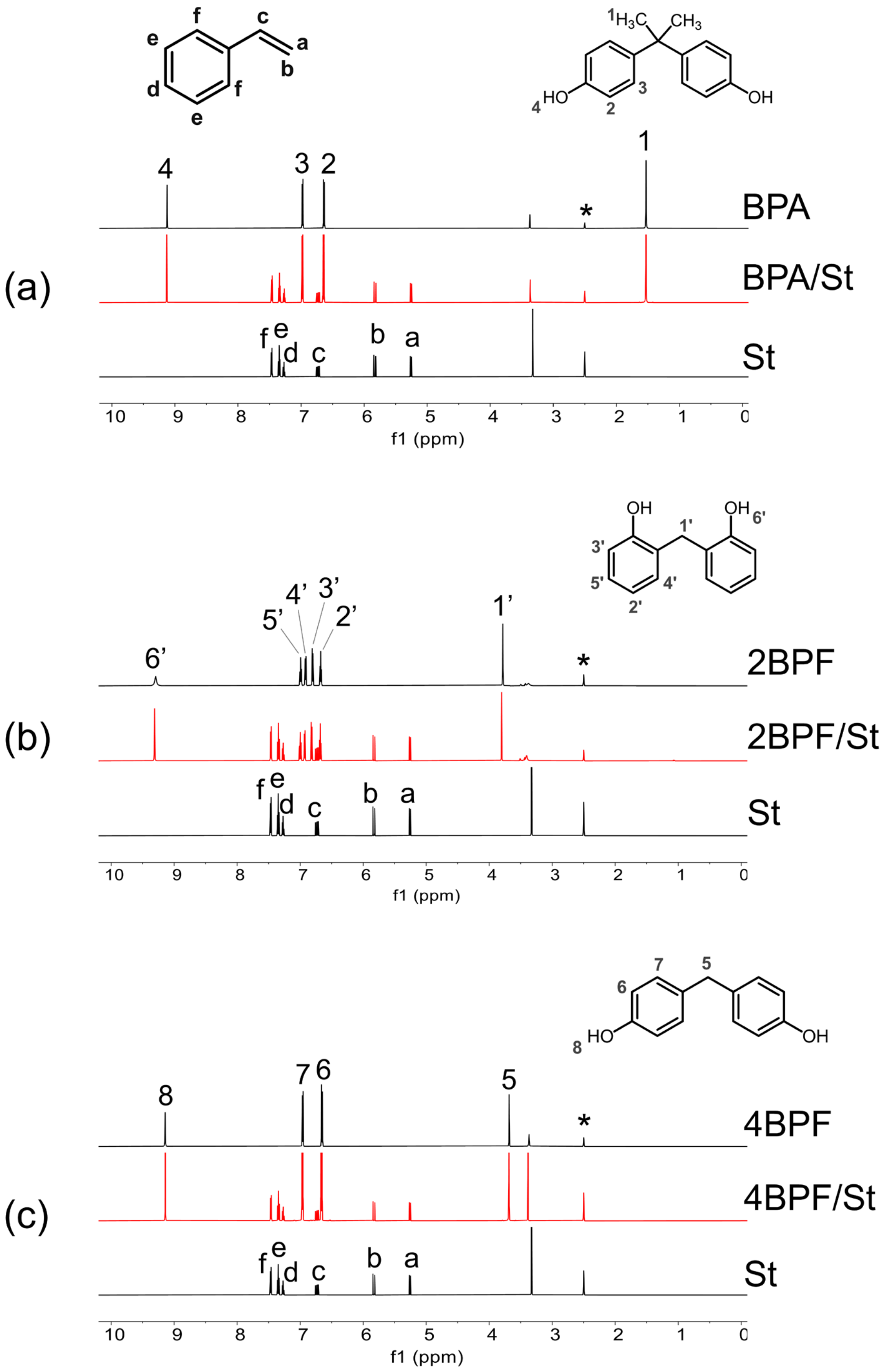

Figure S4. ^{1}H NMR spectra of (a) BPA, (b) 2BPF, and (c) 4BPF upon complexation with St. The molar ratio of bisphenol to St was controlled at 1:2 in DMSO-d_6. The asterisk symbol (*) in the spectra represents the solvent peak of DMSO-d_6.

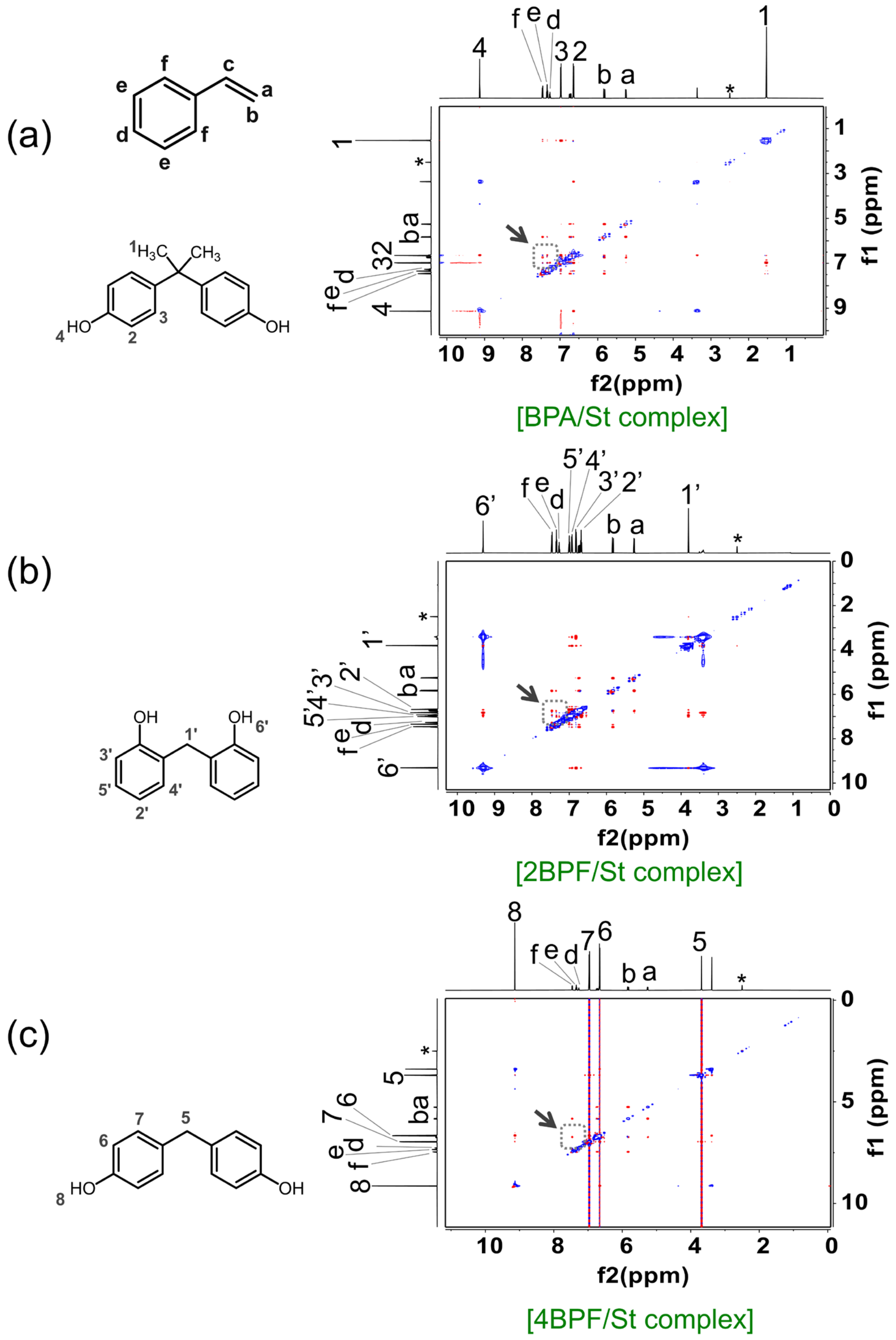

Figure S5. ^{1}H-^{1}H NOESY NMR spectra of (a) BPA/St, (b) 2BPF/St, and (c) 4BPF/St complexes (The black arrows indicate NOE cross peaks of aromatic protons in bisphenol/St). The molar ratio of bisphenol to St was controlled at 1:2 in DMSO-d_6.

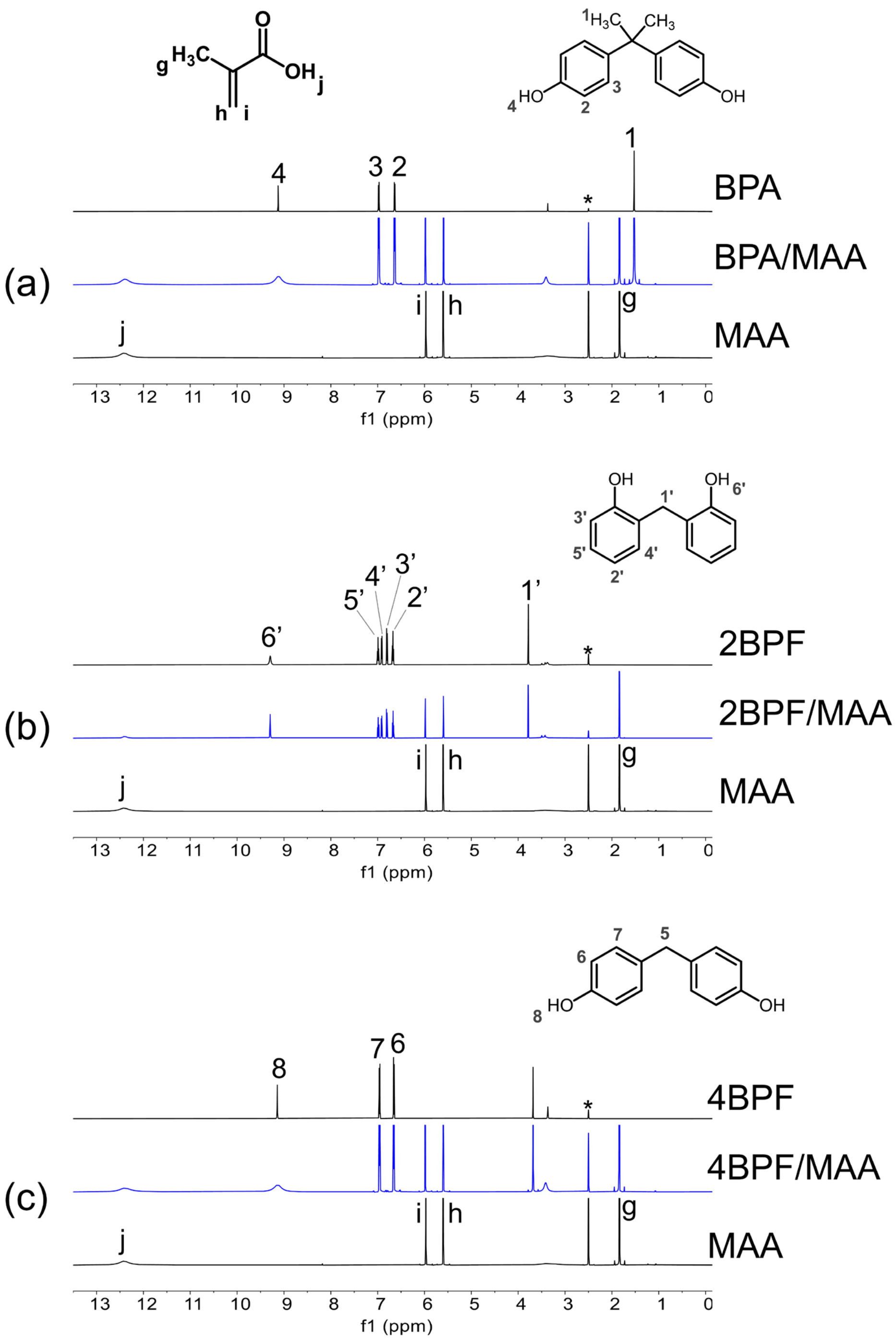

Figure S6. ^{1}H NMR spectra of (a) BPA, (b) 2BPF, and (c) 4BPF upon complexation with MAA. The molar ratio of bisphenol to MAA was controlled at 1:2 in DMSO-d_6. The asterisk symbol (*) in the spectra represents the solvent peak of DMSO-d_6.

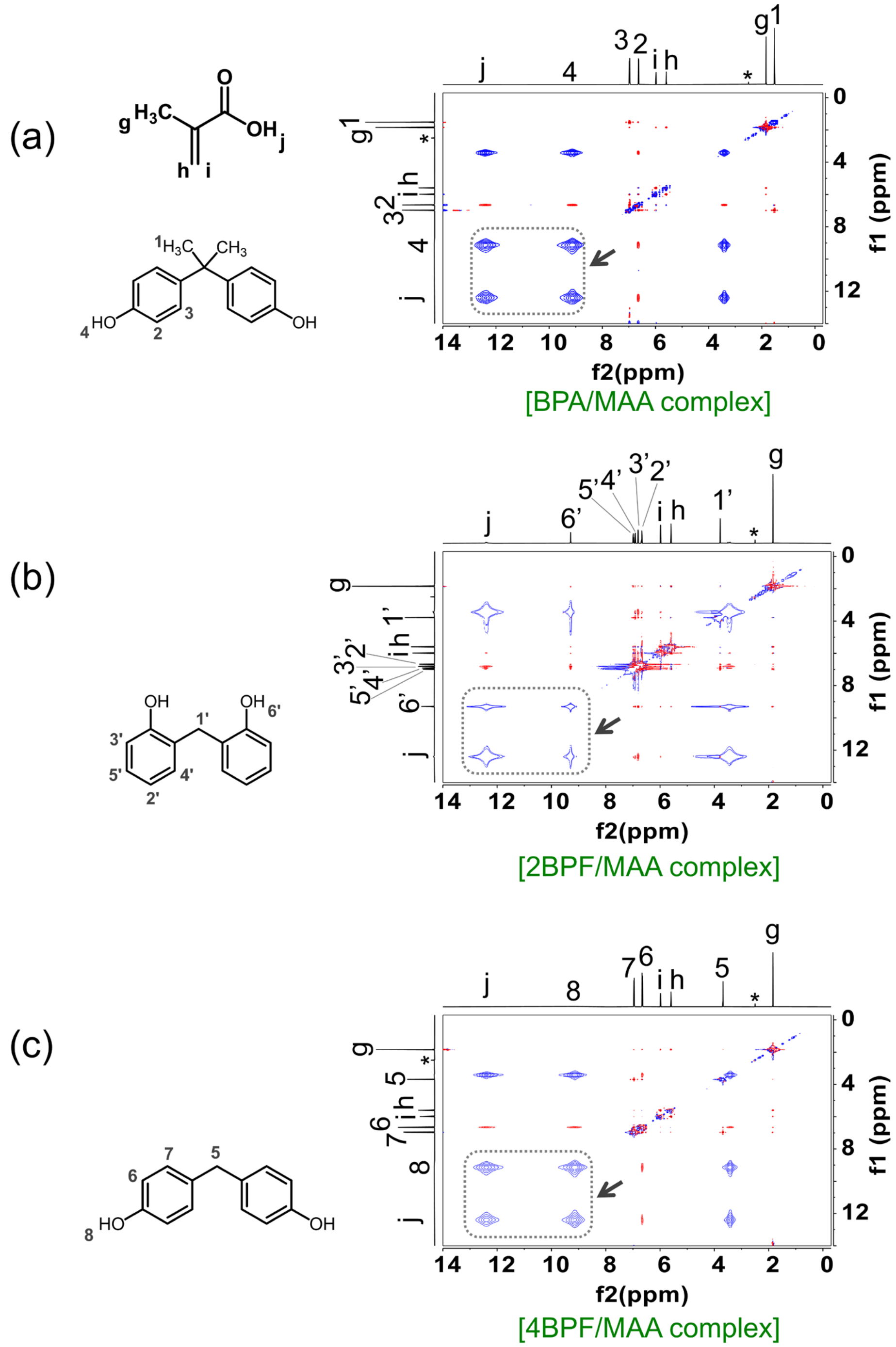

Figure S7. ^1H-^1H NOESY NMR spectra of (a) BPA/MAA, (b) 2BPF/MAA, and (c) 4BPF/MAA complexes (The black arrows indicate the –OH/–COOH proton exchange cross peaks of bisphenols/MAA complexes). The molar ratio of bisphenol to MAA was controlled at 1:2 in DMSO-d_6.

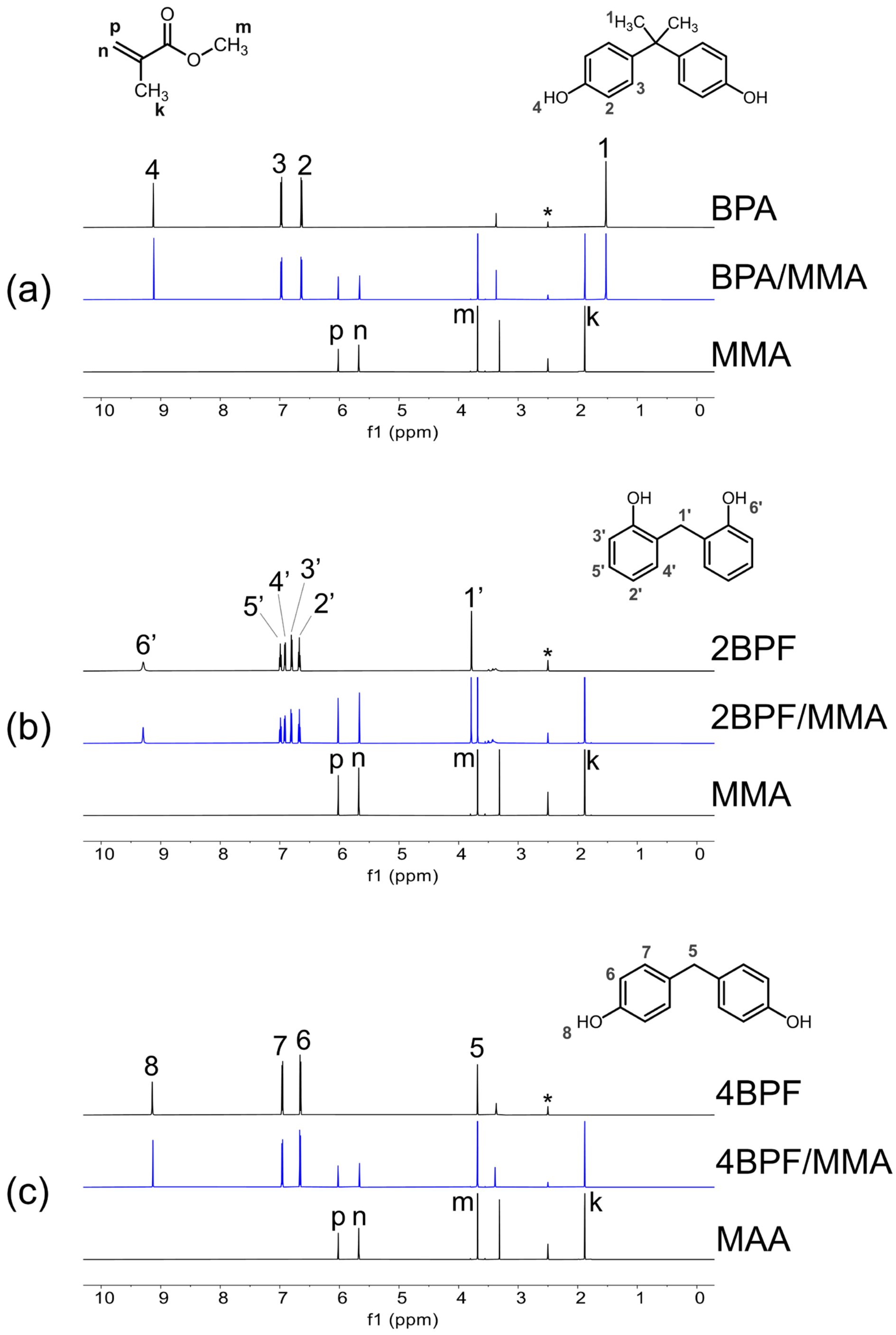

Figure S8. ^{1}H NMR spectra of (a) BPA, (b) 2BPF, and (c) 4BPF upon complex with MMA. The molar ratio of the bisphenol to MMA was controlled at 1:2 in DMSO-d_6. The asterisk symbol (*) in the spectra represents the solvent peak of DMSO-d_6.

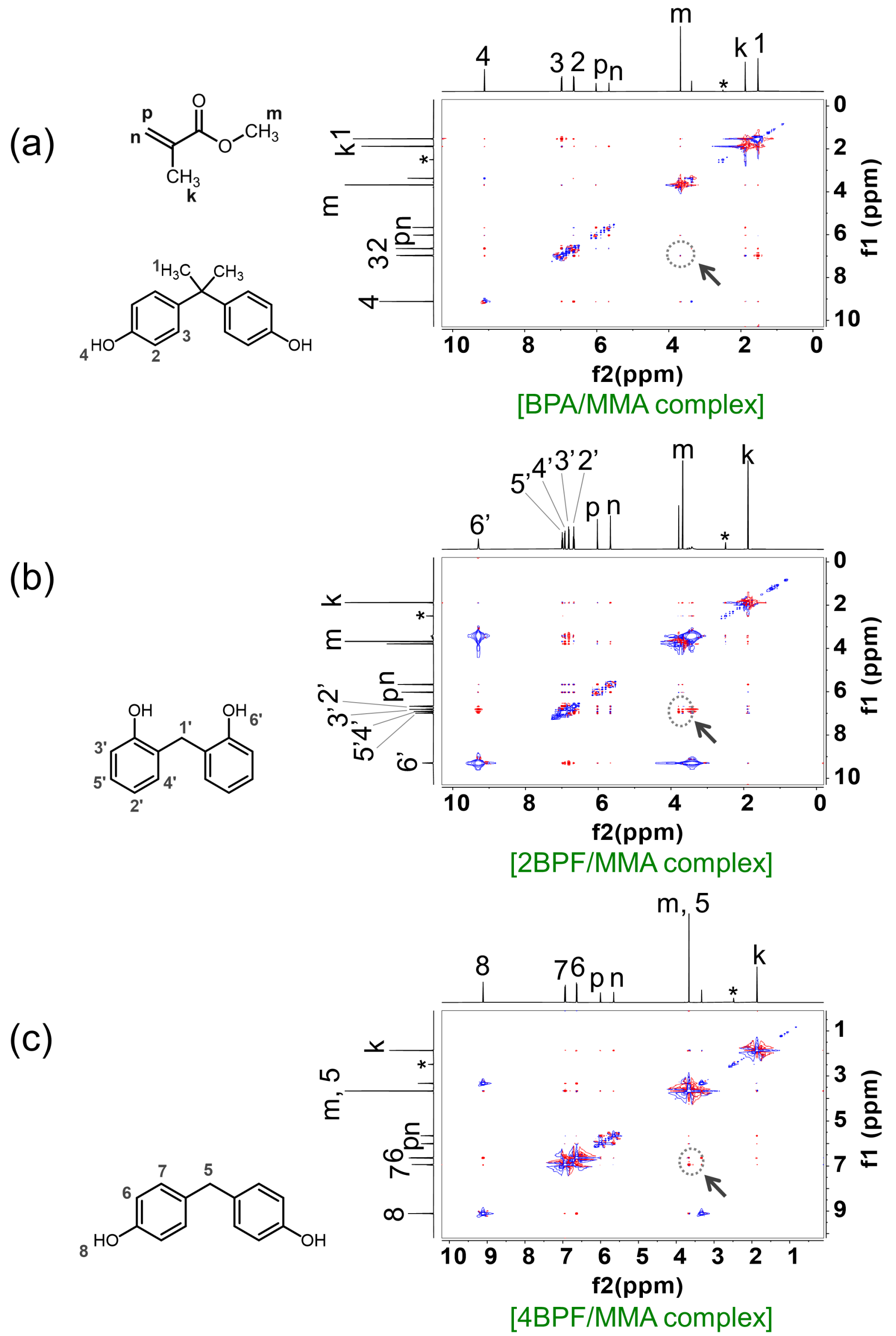

Figure S9. ^1H-^1H NOESY NMR spectra of (a) BPA/MMA, (b) 2BPF/MMA, and (c) 4BPF/MMA complex (The black arrows indicate aromatic rings/–OCH_3 NOE cross peaks of bisphenols/MMA). The molar ratio of the bisphenol to MMA was controlled at 1:2 in DMSO-d_6. The asterisk symbol (*) in the spectra represents the solvent peak of DMSO-d_6.

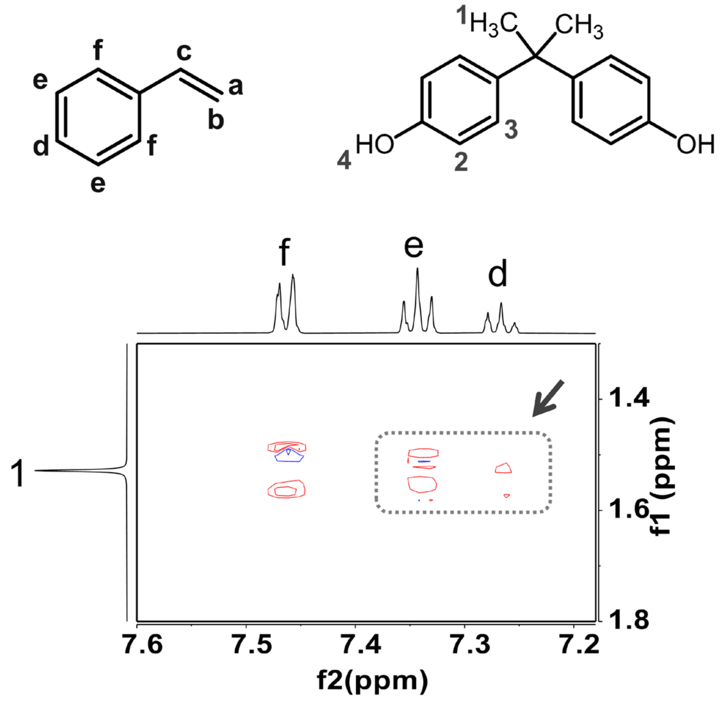

Figure S10. Partial ^1H-^1H NOESY NMR spectra of BPA/St complex. The black arrow points the NOE cross-peaks between –CH_3 proton of BPA and aromatic protons of St. The molar ratio of the bisphenol to St was controlled at 1:2 in DMSO-d_6.

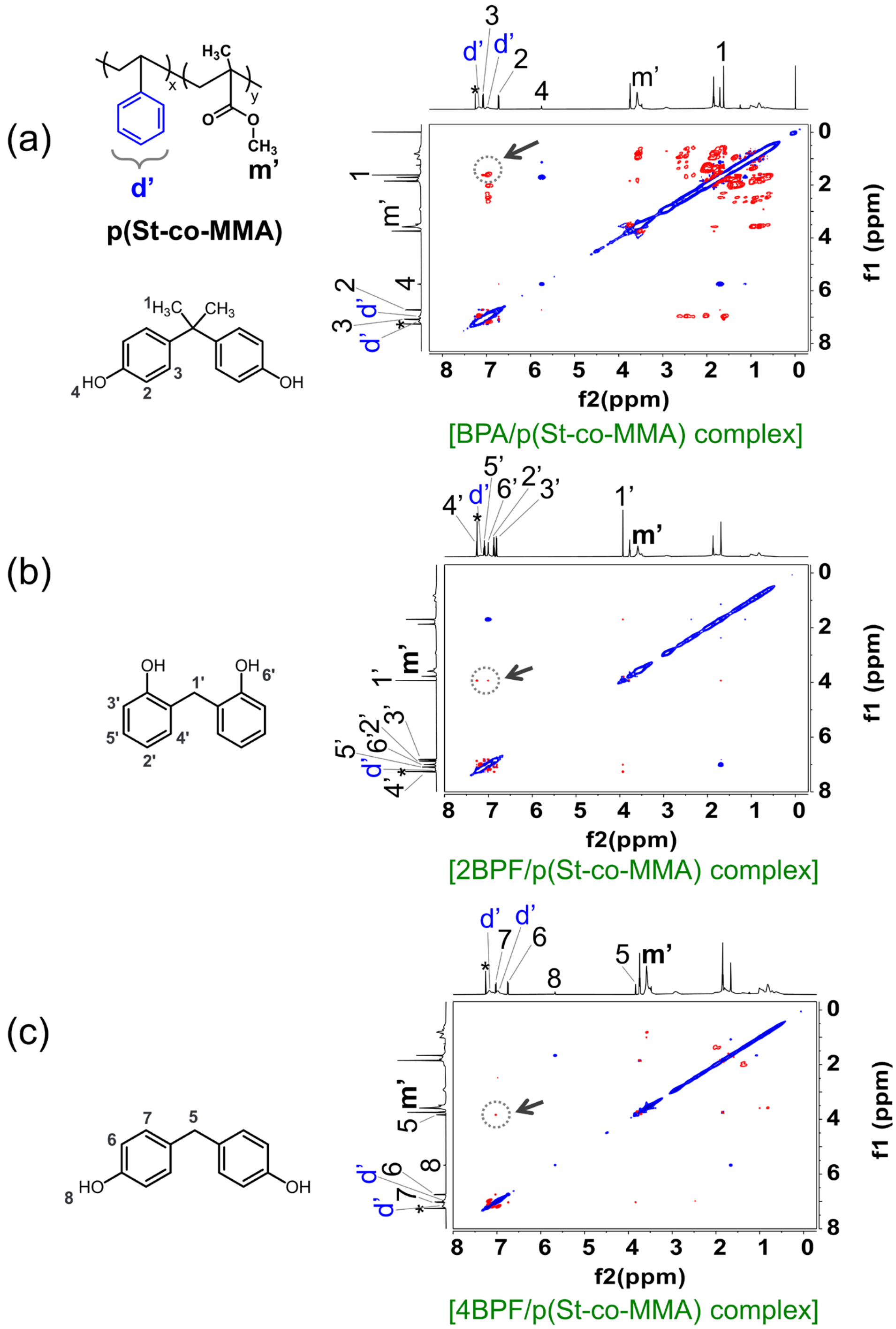

Figure S11. ^1H-^1H NOESY NMR spectra of p(St-co-MMA) upon association with (a) BPA, (b) 2BPF, and (c) 4BPF in $CDCl_3$. The asterisk symbol (*) represents the solvent peak of $CDCl_3$.

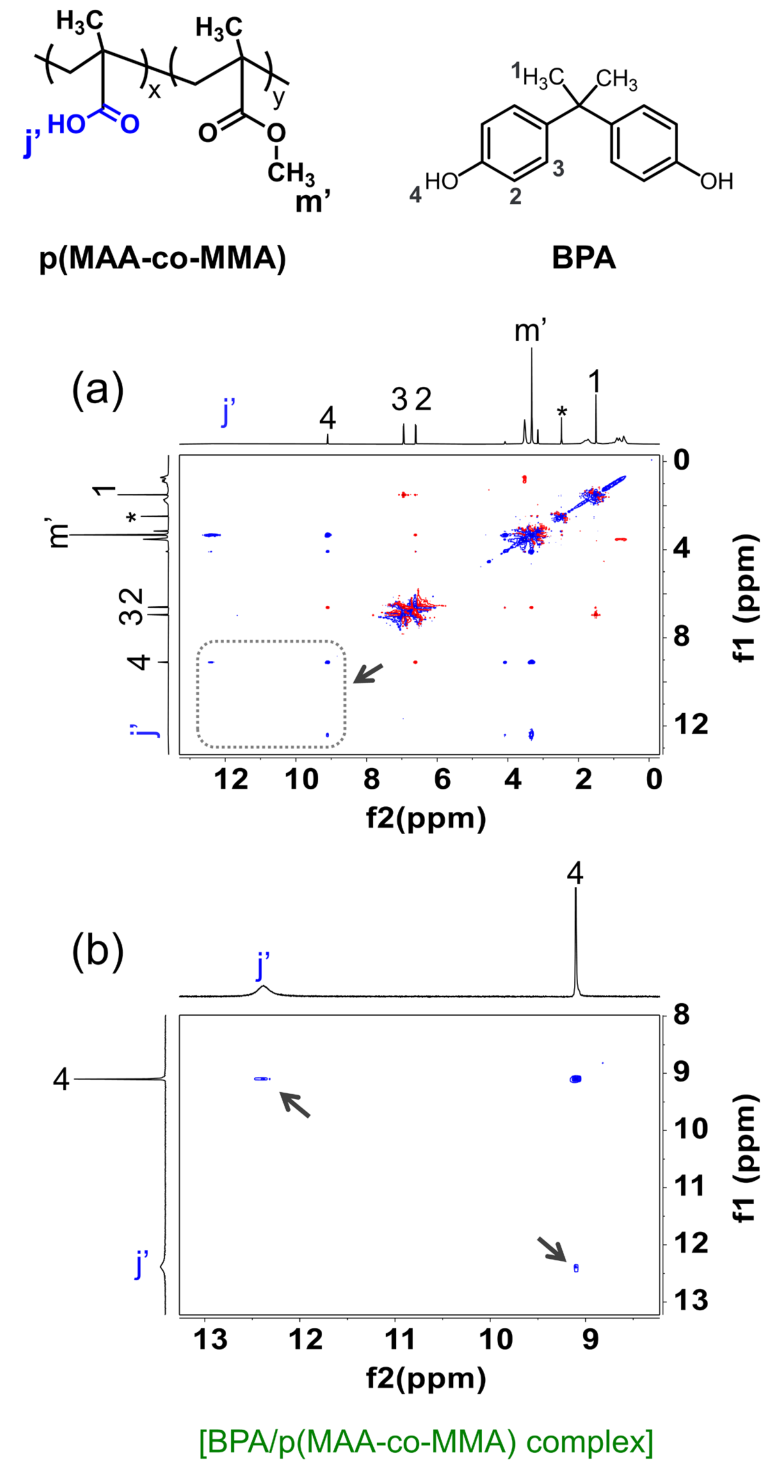

Figure S12. ^{1}H-^{1}H NOESY NMR spectra of p(MAA-co-MMA) upon association with (a) BPA in DMSO-d_6 and (b) the enlarged spectra within the boxed region in S12a. The asterisk symbol (*) represents the solvent peak of DMSO-d_6.

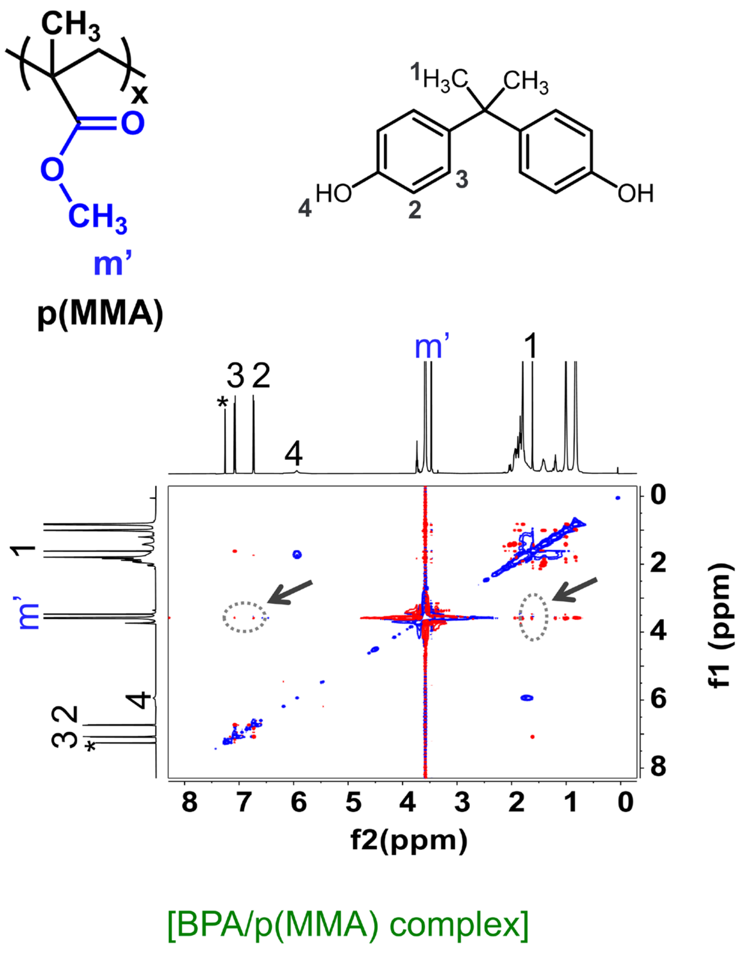

Figure S13. ^1H-^1H NOESY NMR spectra of p(MMA) upon association with BPA in $CDCl_3$. The asterisk symbol (*) represents the solvent peak of $CDCl_3$.
